# Viscoelastic Response in Hydrous Polymers: The Role of Hydrogen Bonds and Microstructure

**DOI:** 10.1021/acs.nanolett.4c00556

**Published:** 2024-03-12

**Authors:** Wenbo Chen, Philip Biehl, Caoxing Huang, Kai Zhang

**Affiliations:** † Sustainable Materials and Chemistry, Department of Wood Technology and Wood-based Composites, University of Göttingen, Büsgenweg 4, Göttingen 37077, Germany; ‡ Co-Innovation Center of Efficient Processing and Utilization of Forest Resources, College of Chemical Engineering, Nanjing Forestry University, Nanjing, Jiangsu 210037, China

**Keywords:** cellulose, viscoelastic response, nanoparticle, hydrogen bond, noncovalent interaction, strain hardening

## Abstract

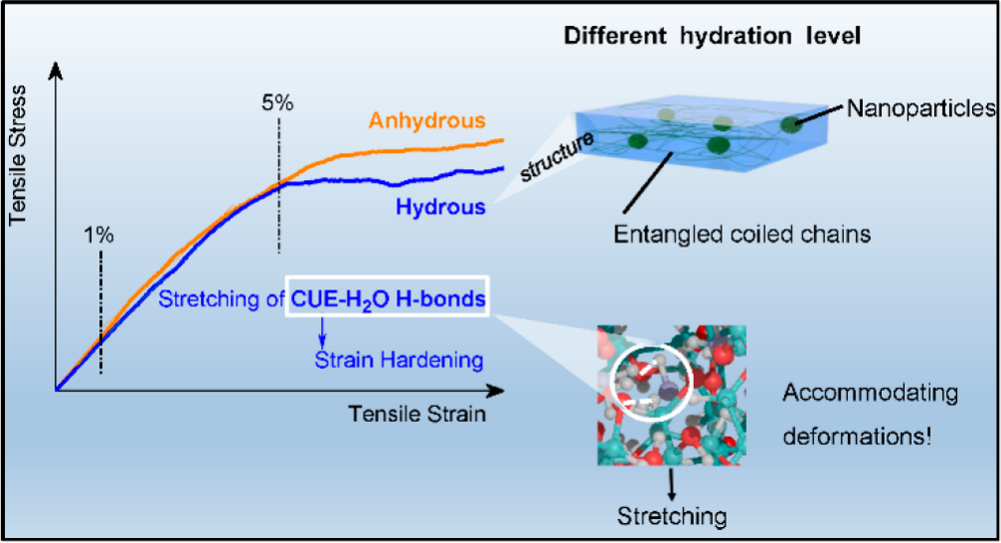

Water responsive polymers represent a remarkable group of soft materials, acting as a laboratory for diverse water responsive physical phenomena and cutting-edge biology–electronics interfaces. We report on peculiarly distinctive viscoelastic behaviors of the biobased water responsive polymer cellulose 10-undecenoyl ester, while biobased regenerated cellulose displays stronger hydroplastic behaviors. We discovered a novel hydrous deformation mechanism involving the stretching of hydrogen bonds mediated by hydroxyl groups and water molecules, serving as a crucial factor in accommodating deformations. In parallel, the microstructure of cellulose 10-undecenoyl ester with unique coexisting nanoparticles and a continuous phase of entangled chains is mechanically resilient in the anhydrous state but enhances structural stiffness in the hydrous state. This variation arises from a different hydration level within the hydrous microstructure. Such a fundamental discovery offers valuable insights into the connection between the microscopic physical properties that can be influenced by water and the corresponding viscoelastic responses, extending its applicability to a wide range of hygroscopic materials.

Hydroplastics represent a cutting-edge field at the intersection of materials science and environmental sustainability. The underlying polymers made from sustainable cellulosic compounds make up a class of amphiphilic materials, characterized by their tunable water responsive mechanical properties.^1,2^ The presence of water in hydroplastic polymers allows controlled deformations across a diverse mechanical spectrum. Understanding the varied mechanical influence of water based on the microscopic physical nature of hydroplastic polymers becomes crucial in appreciating their response to applied stress. As a result, this ability can be effectively used in a wide array of applications, like intelligent actuators and sensors.^3−5^

Despite numerous efforts to investigate the influence of water on the properties of hygroscopic materials, including its impact on their mechanical properties,^6−9^ fundamental insights into the relationship between the water-related microscopic physical nature and macroscopic viscoelastic responses is still unclear. The demonstrated reversible water responsive mechanical change is closely connected to the dynamic breaking and reforming of a high-density hydrogen-bond (H-bond) network. This forms the foundation for a diverse range of viscoelastic responsive mechanics.^10−12^ In parallel, it is also crucial to recognize that the mechanical response is intricately dependent on the material’s structure.^13−15^ The mechanical response is determined by the microstructure of the material, and the applied stress induces structural changes, which in turn will change the overall mechanical properties.^16,17^

In this work, we performed comprehensive static and dynamic mechanical experiments to capture the viscoelastic responsive portfolio of a hydroplastic polymer, cellulose 10-undecenoyl ester (CUE_0.3_), in anhydrous and hydrous states. We discovered that its physical nature, specifically the dynamic H-bond system and microstructure, plays a crucial role in shaping the viscoelastic behaviors that can be influenced by water. To gain deeper insights into the microscopic mechanisms, we explored the viscoelastic response of a fundamentally similar hydroplastic polymer, regenerated cellulose (RC), as a comparison. Surprisingly, we found a distinctive effect of water on their viscoelastic responsive behaviors. To understand the changes in the H-bonds, the microstructure, and the origins of the mechanical properties at the atomic level, we performed extensive all-atom molecular dynamics (MD) simulations by using the polymer consistent force field (PCFF) and reactive force field (ReaxFF).

## Multiscale Structure and Molecular Interactions in Hydrous CUE_0.3_ Membranes

**Figure 1:**
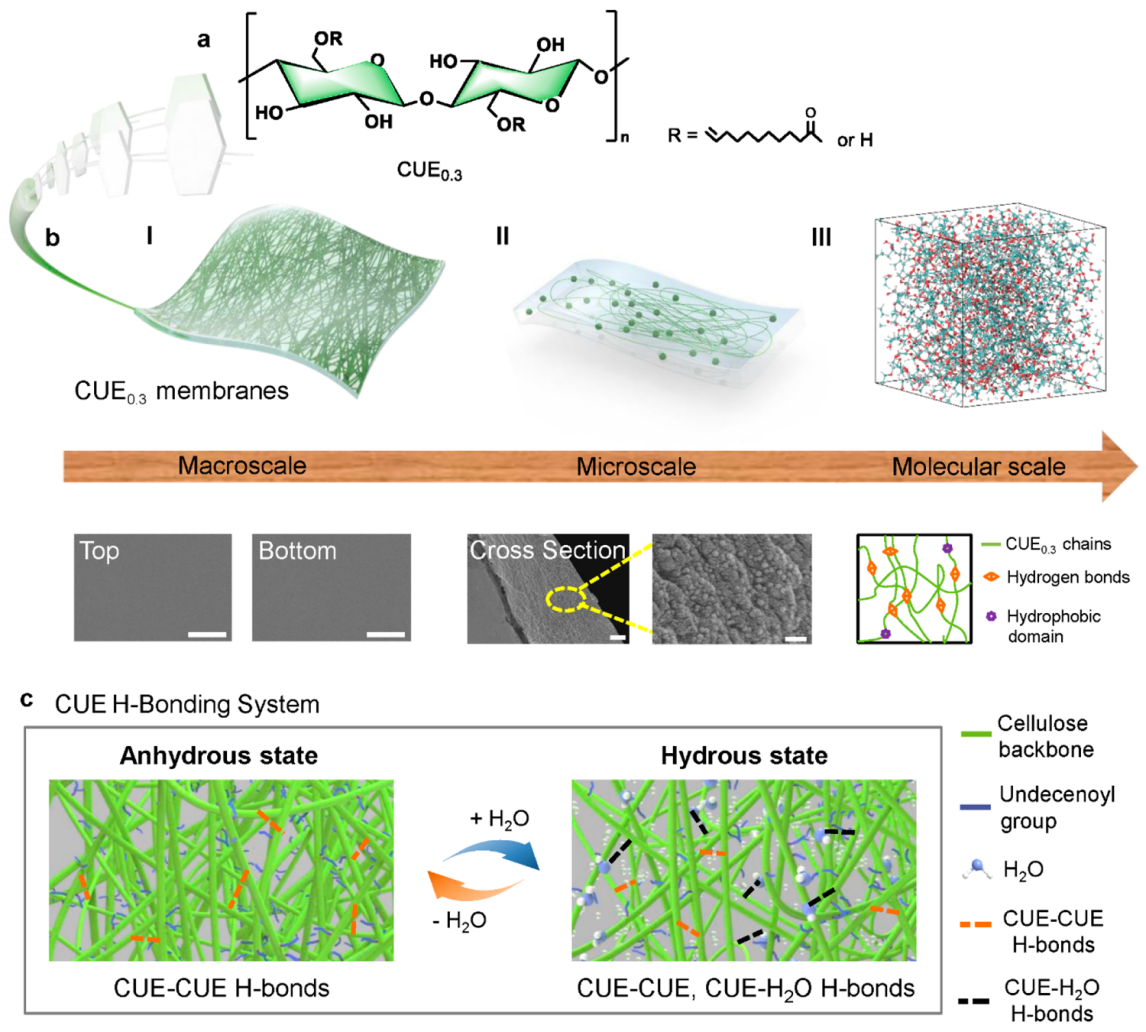
(a) Chemical structure of CUE_0.3_. (b) Multiscale structure of the CUE_0.3_ membrane. Visualized diagram of their (I) macroscale membranes, (II) microscale cross-section morphology, (III) molecular scale CUE_0.3_ model, and aggregated polymer domains with various interactions. The cyan, white, and red colors represent carbon, hydrogen, and oxygen atoms in the model, respectively. The oxygen atom in the H_2_O molecule is colored purple for the sake of clarity. Correspondingly, scanning electron microscopy (SEM) images showing the top and bottom surface (scale bars of 5 μm), the cross section (scale bar of 2 μm), and the enlarged cross section of the hydrous CUE_0.3_ membranes (scale bar of 300 nm). (c) Illustration of the distinct H-bond system for CUE_0.3_ in the anhydrous and hydrous states.

The CUE_0.3_ polymer was prepared by esterifying microcrystalline cellulose with 10-undecenoyl chloride in an *N,N*-dimethylacetamide (DMAc)/lithium chloride (LiCl) mixture. Figure 1a demonstrates its chemical structure as verified by ^13^C and ^1^H nuclear magnetic resonance (NMR) and Fourier-transform infrared (FTIR) spectroscopy (Figures S1 and S2). The degree of substitution was calculated to be 0.3 on the basis of elemental analysis. By contrast, the RC polymer was prepared by dissolving microcrystalline cellulose in a DMAc/LiCl mixture. The as-prepared CUE_0.3_ membranes were manufactured via a facile solvent casting method (Figure 1b), while the RC membranes were manufactured via a classical phase separation process after air drying. These as-prepared membranes are flat and highly transparent with a low haze (Table S1). Surprisingly, CUE_0.3_ and RC membranes (Figure S3) showed similar smooth surfaces on the top and bottom layers, while the cross section of CUE_0.3_ membranes revealed nanoparticles within a continuous matrix.

The presence of water can disrupt the H-bond network within anhydrous CUE_0.3_, resulting in the coexistence of CUE–CUE and CUE–H_2_O H-bonds (Figure 1c). Such alterations substantially influence the material’s mechanical properties and enable a distinctive viscoelastic response.

## Viscoelastic Response in Anhydrous and Hydrous CUE_0.3_ Membranes

**Figure 2:**
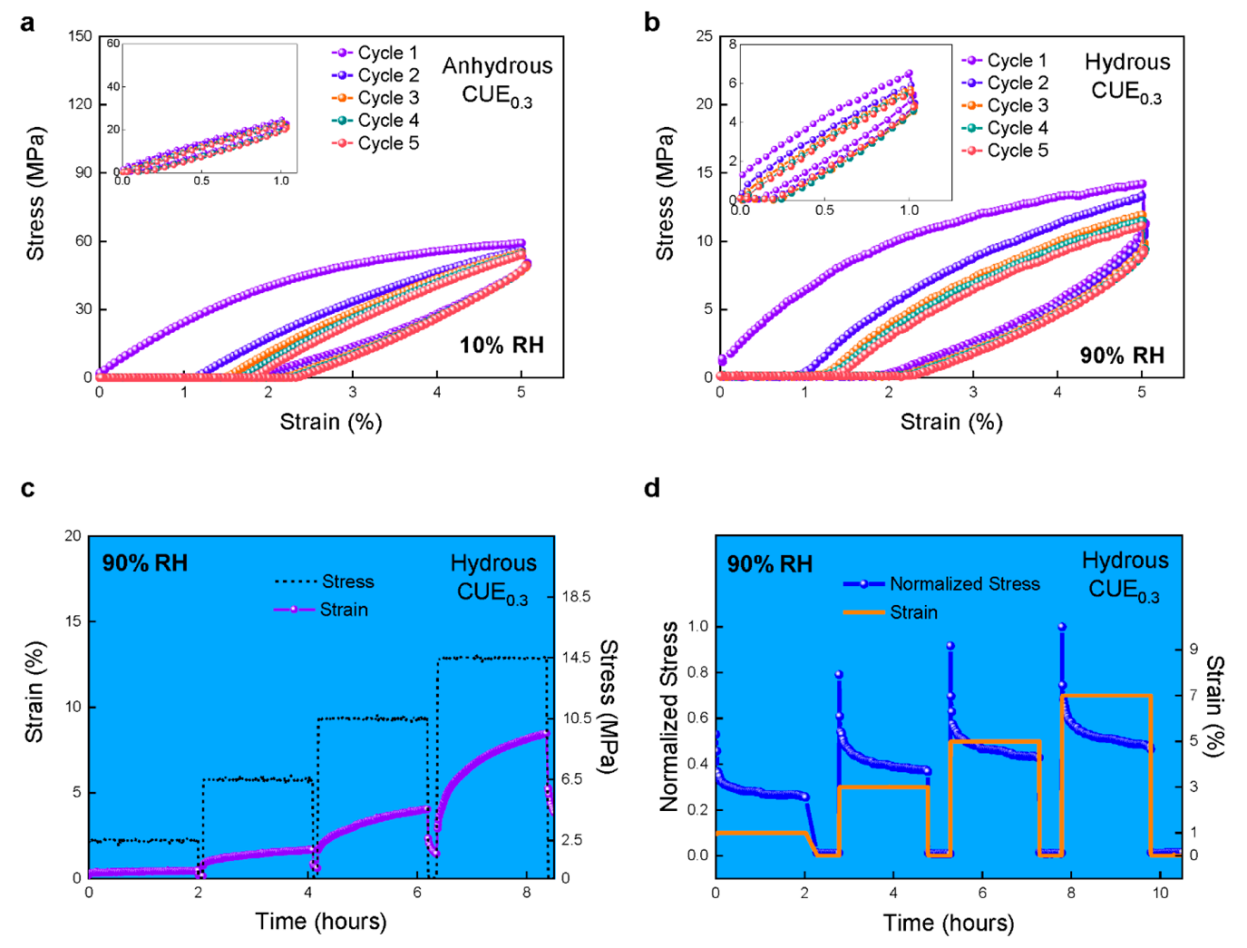
Experimental cyclic stress–strain responses up to 5% strain of (a) anhydrous CUE_0.3_ membranes at 10% RH and (b) hydrous CUE_0.3_ membranes at 90% RH. Insets show cyclic stress–strain responses of the same membranes at 1% strain. (c) Consecutive plasticity mechano-creep experiments of hydrous CUE_0.3_ membranes upon step loading and partial unloading cycles at a constant 90% RH. (d) Consecutive plasticity mechano-stress relaxation experiments of hydrous CUE_0.3_ membranes upon loading and unloading cycles at a constant 90% RH. The stress was calculated by dividing the force by the cross section of the specimen. The strain (percent) was defined as (*L* – *L*_0_)/*L*_0_ × 100%, where *L* is the instantaneous length and *L*_0_ is the initial length of the specimen.

To investigate the effect of equilibrated water on the viscoelastic response, we analyzed cyclic stress–strain curves of CUE_0.3_ and RC membranes in the anhydrous and hydrous states. To avoid destructive plasticity, the membranes were cyclically stretched with an elastic strain of 1% and 5% for five cycles. As shown in Figure 2a, anhydrous CUE_0.3_ membranes at 10% relative humidity (RH) demonstrated a hysteresis loop in cycle 1, indicating substantial energy dissipation due to the disruption of multiple H-bonds during stretching.^12,18,19^ At 1% strain, the loading and unloading curves in each cycle followed similar paths, revealing a small elastic hysteresis. At 5% strain, the energy dissipation in cycle 2 was smaller than that in cycle 1. Disrupted H-bonds did not undergo sufficient restoration to return to their initial state amid sequential stretching. The loading curve exhibited approximate linear elastic responses after four cycles, while the unloading curve exhibited a minor change with an increase in the number of cycles. The nanoparticles with a high polymer chain density within anhydrous CUE_0.3_ membranes should have effectively restricted the chain movement and slippage, leading to enhanced mechanical resilience while they were being stretched within 5% strain.^20^

Hydrous CUE_0.3_ membranes at 90% RH exhibited obvious elastic hysteresis during cyclic stretching at both 1% and 5% strain (Figure 2b). Although the external high-RH setting can induce plasticization on the membrane surface, the impact on the viscoelastic response was negligible due to its reversible nature (Figure S4). During cycling tests, a progressive decrease in stress by the same strain occurs, along with a trend similar to that of viscous strain hardening.^21^ Surprisingly, the initial slope of each unloading curve was steep, indicating high elastic stiffness despite the obvious viscous behavior.^22−24^ Notably, the materials underwent viscoelastic deformation during each cycle for energy dissipation. This finding is in agreement with water-induced plasticization with the substantial elimination of entanglements in amorphous polymers, while the remaining trapped entanglements can still contribute to the mechanical resilience, as elucidated by Rubinstein and Colby.^25^ In contrast, RC membranes with a primitive cellulosic H-bond system exhibited a 5% strain plastic response in the anhydrous state, while they demonstrated a substantial entropy elastic response in the hydrous state (Figure S5). This implies a distinctive effect of water on the viscoelastic response of the CUE_0.3_ and RC membranes.

Surprisingly, dynamic mechanical thermal analysis (DMTA) demonstrated that CUE_0.3_ and RC membranes shared a similar RH-dependent dynamic viscoelasticity and chain mobility, as well as a robust structural integrity and long-term mechanical durability (Figures S6–S8). The results suggest that (1) when hydrous CUE_0.3_ and RC polymer chain segments start to move upon stretching within elastic limit strain,^26^ there should be no substantial difference in their internal friction and (2) hydrous RC can exhibit a higher elastic energy^27^ and demonstrated therefore an entropic elasticity.

To further validate the time-dependent dynamic viscoelasticity in hydrous CUE_0.3_ and RC membranes, consecutive plasticity mechano-creep measurements at a constant 90% RH were analyzed (Figure 2c). Hydrous CUE_0.3_ exhibited a notable viscoelastic behavior, contrasting with the highly elastic behaviors observed in hydrous RC (Figure 2c, Figure S5c, and Table S4). This behavior is characterized by the time-dependent nonlinear strain recovery when the applied stress is withdrawn, as well as the weakening elastic response of the material caused by the residual strain in each cycle.^28,29^ Therefore, the plastic deformation occurs due to the decreasing capacity of nanoparticles upon hindering of the mobility of polymer chains above the elastic stress threshold (herein 10.5 MPa).^30^ Unlike RC membranes, the stresses in hydrous CUE_0.3_ can be relaxed to a certain value in all cycles (Figure 2d), and CUE_0.3_ membranes exhibited a superior capacity to retain the residual stresses by strains of <5%, which could be attributed to the significant impact of nanoparticles (Figure S5d and Table S5). Therefore, it is reasonable to elucidate the distinctive viscoelastic response of hydrous CUE_0.3_ and RC membranes from a morphological perspective, considering that unique nanoparticles within the CUE_0.3_ membrane can dynamically contribute to its mechanical behavior when in contact with water.

## Water-Induced Transition of the Microstructure and H-Bond System in CUE_0.3_ Membranes

**Figure 3:**
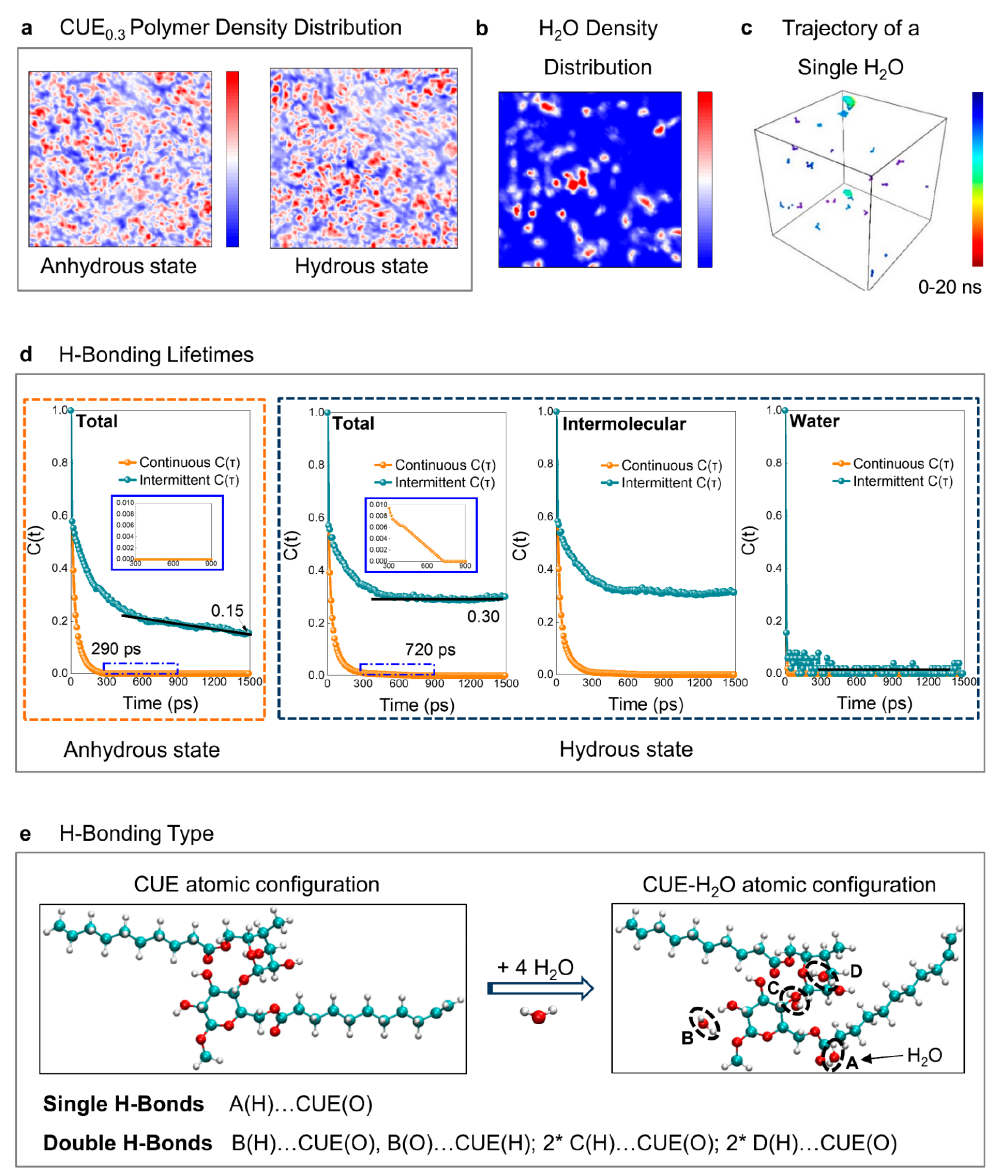
(a) Averaged CUE_0.3_ polymer density distribution profile at equilibrium anhydrous and hydrous states in the MD box. (b) Averaged H_2_O density distribution profile in the equilibrium hydrous state in the MD box. The red color denotes the regions of space most likely to be occupied, while the blue space is not occupied. (c) Trajectory of a single H_2_O molecule during 20 ns in the MD box at equilibrium. The color spectrum from red to blue marks the evolution of time. (d) Continuous and intermittent H-bond lifetime autocorrelation function of the CUE_0.3_ polymer in the anhydrous and hydrous states. In the inset, a blue solid-line frame shows the enlarged time period from 300 to 900 ps. (e) Optimized stable atomic configuration and H-bond types on one repeating unit of CUE and the CUE–H_2_O model based on the atoms in molecules theory. The letters A–D in the diagram represent the H_2_O molecules.

The unique water-related viscoelastic response of CUE_0.3_ was investigated with regard to its enormous impact on microstructures and H-bond transitions. Given our focus on equilibrium rather than diverse conditions (e.g., different water content and diffusion phase^31^), we conducted MD simulations on samples in both anhydrous and hydrous states. RC serves as a basis for comparative analysis (Figure S9). To ensure accurate results, the water content of the hydrous samples in the MD simulations was obtained from DVS measurements (Figure S10). The variations in the water-triggered viscoelastic response of the CUE_0.3_ polymer are intended to be linked to the polymer and water density distribution profile of the CUE_0.3_ model (Figure 3a,b).

The CUE_0.3_ polymer exhibited a uniform density distribution (Figure 3a). The blue areas correspond to regions in which the polymer density is lower than that of the red areas, where a large number of micronanopores should be presented. Upon hydration of the polymer chains, blue regions were not observed to interconnect into larger areas. Therefore, limited unspinning of the polymer chains occurred despite their fewer interactions. Moreover, water exhibited a relatively uniform density distribution (Figure 3b). The large blue region represents those unavailable for water molecules and therefore not for their transport. In addition, a single water molecule could access most of the system within 20 ns (Figure 3c). Thus, a large portion of adsorbed water is trapped through the formation of restricted and dynamic H-bonds with the abundant hydroxyl groups on the polymer chains. Although some water molecules with unstable H-bonds may persist in the system, they are more likely to move through the system rather than accumulating at a specific point for water clusters. The statement is coincident with the DVS results (Figure S10).

Dynamic interactions between water molecules and CUE_0.3_ polymer chains in the system are evident through the fluctuations in both continuous and intermittent H-bond lifetimes (Figure 3d). The addition of water molecules strengthens the stability of the H-bond network in CUE_0.3_. The degree of decay of the continuous H-bond lifetime function in hydrous CUE_0.3_ was dramatically decreased by a factor of 2.5 compared to that in anhydrous CUE_0.3_, indicating the recently established more stable and persistent H-bond network. In parallel, the rate of decay of intermittent H-bonds in hydrous CUE_0.3_ rapidly approaches zero and maintains a constant intermittent *C*(*t*) over an extended time scale, while anhydrous CUE_0.3_ consistently increases with simulation time. Moreover, the intermittent *C*(*t*) value of hydrous CUE_0.3_ at 1490 ps was twice that of anhydrous CUE_0.3_, representing longer intermittent H-bond lifetimes. The intermolecular H-bond lifetime function in hydrous CUE_0.3_ shows that this favorable variation was attributed to the continuous formation and breakage of water-mediated intermolecular H-bonds, thereby stabilizing CUE_0.3_. In contrast, the continuous and intermittent H-bond lifetime functions among water molecules were negligibly short due to their highly dynamic nature. Furthermore, the typical H-bonds between CUE_0.3_ and H_2_O involved one single and three double H-bonds^32^ (Figure 3e). The quantitative H-bond energy in one repeating unit of the CUE–H_2_O model was fitted by calculating the critical point electron density via the atoms in molecules theory (Figure S11 and Table S3).

## Molecular Dynamics Understanding of the Mechanical Mechanism in Hydrous CUE_0.3_ Membranes

**Figure 4:**
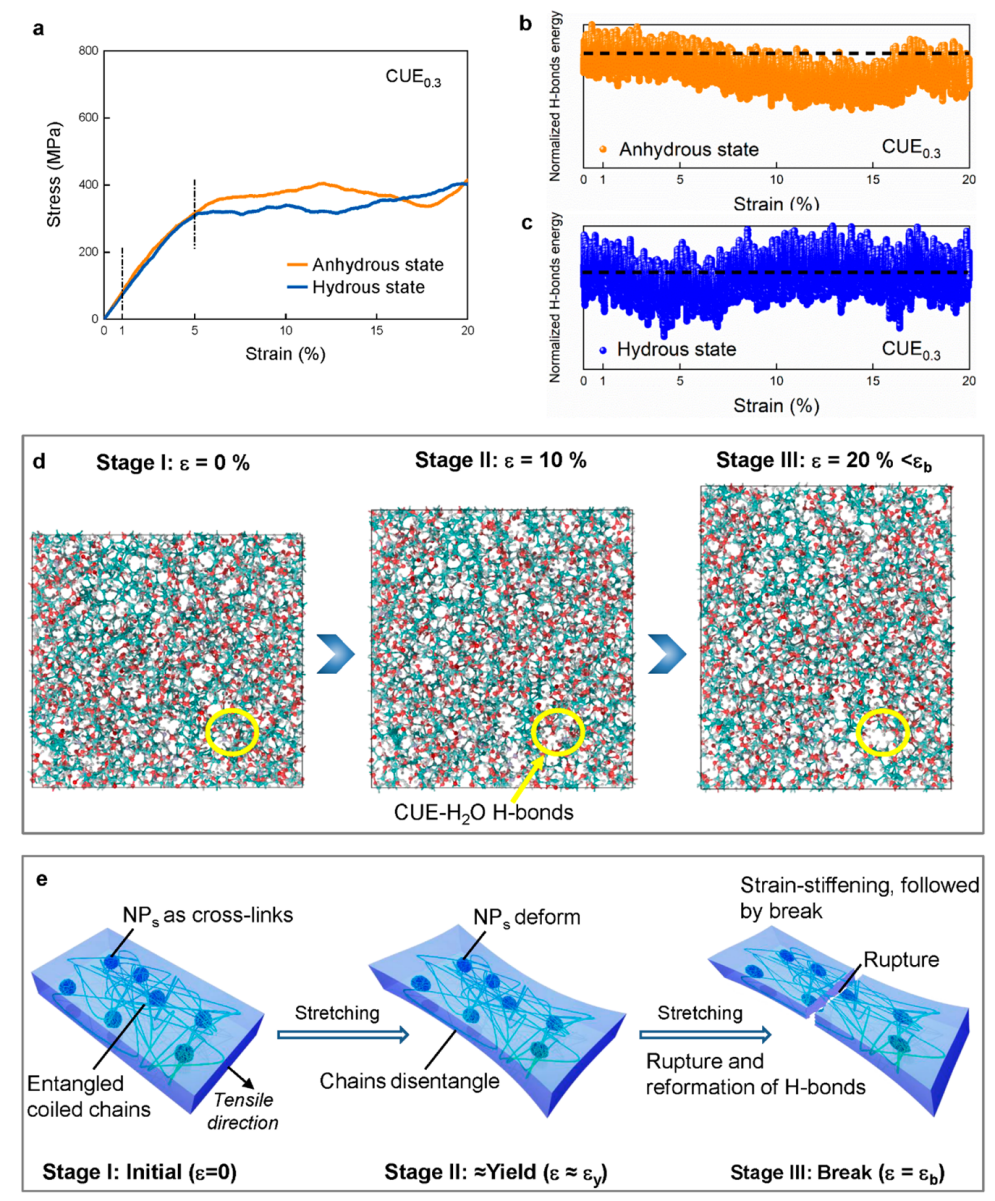
(a) MD-simulated stress–strain curves. Experimental stress–strain curves in Figure S12. Changes in the normalized H-bond energy in (b) anhydrous and (c) hydrous CUE_0.3_ as a function of strain. (d) Snapshots for the MD-simulated movements of hydrous CUE_0.3_ polymer chains with an increase in strain. Enlarged local snapshots in Figure S13. (e) Schematic illustrations of mechanical mechanisms upon stretching. NP_s_ means the nanoparticles, and ε stands for each different strain.

In addition to experimental evidence, MD simulations on custom-built models under tension were conducted to better understand the mechanical mechanism between polymer chains and water molecules from a perspective of H-bonds and microstructure. The MD-simulated stress–strain curves can predict the experimental ones well for both CUE_0.3_ and RC despite slight deviations (Figures S14 and S15 for RC). The hydrous CUE_0.3_ model exhibited mechanical characteristics of low strength, high strain hardening, and similar Young’s modulus compared to those of the anhydrous CUE_0.3_ model (Figure 4a). This result agrees with findings from the corresponding tensile experiments. The differences in stress–strain values between experiments and simulations primarily exist for two reasons. (1) There is a substantial difference in molecular friction between all-atom molecular models used in simulations and actual polymers. In MD simulations, failure primarily occurs while chains are being pulled out, while the limited computing resources prevent the observation of bond energies that are large enough to cause the disruption of chains. Under the experimental conditions, both failure modes, chain pullout and scission, play a role and mechanically induced failure is strongly dependent on defects.^33,34^ (2) The modulus values strongly depend on the strain rate during tensile tests.^35^ Nevertheless, our model provides accurate estimations of the mechanical events upon stretching, highlighting the role of the H-bond network in determining the overall structural properties, as discussed above.

Panels b and c of Figure 4 plot the variation of the H-bond energy in anhydrous and hydrous CUE_0.3_ as a function of 20% tensile strain. Compared to that of anhydrous CUE_0.3_, the H-bond energy of hydrous CUE_0.3_ initially decreases during the early strain stage and then increases and stabilizes with larger strains. CUE–CUE H-bonds are considered to withstand the initial deformation, yet their presence is observed to be lower in hydrous CUE_0.3_ than in anhydrous CUE_0.3_. Therefore, the H-bond energy stability of hydrous CUE_0.3_ is particularly sensitive to a change in strain. As the strain increases, the CUE–H_2_O H-bonds begin to compete with the CUE–CUE H-bonds, and the rebound in the H-bond energy signifies the increasing influence of the CUE–H_2_O H-bonds in the mechanical response. A pronounced increase also in Coulomb energy was observed for hydrous CUE_0.3_, but its development trend was similar upon stretching (Figure S16a,b). Therefore, the presence of water preserves to a large extent the atomistic packing during the mechanical deformation, which does not influence the modulus.^36^ Moreover, the torsional angular energy of hydrous CUE_0.3_ remained the same as that in anhydrous CUE_0.3_. Instead, it slightly increased with strain (Figure S16c,d). Thus, CUE–H_2_O H-bonds constrain the structural changes in CUE_0.3_.^37^ The macroscopic deformation of anhydrous CUE_0.3_ essentially translates to H-bond stretching (Figure S17). With respect to hydrous CUE_0.3_, H-bond stretching is not the sole mechanism for accommodating deformations when a certain number of waters adsorb at the -OH sites. Because most water molecules possess rotational degrees of freedom, they can also reorient themselves and thus quickly break and reform new CUE–H_2_O H-bonds in response to external load. Therefore, the strain in H-bonds could be minimized (Figure 4d) and the brittle failure of the material due to CUE–CUE H-bond breakage is less likely.

In addition, Figure 4e emphasizes the impact of the microstructure on the macroscopic mechanical response in hydrous CUE_0.3_. The substantially different mechanical contribution of nanoparticles to the mechanical response depends on the presence or absence of water. Within the range of elastic strain, they provide mechanical resilience in the anhydrous state, thereby enhancing the material’s fatigue resistance, while in the hydrous state, they contribute to structural stiffness, improving the material’s creep resistance. This variation arises from the different hydration level between the coexisting nanoparticles and entangled chains within the hydrous microstructure. Beyond elastic strain, such nanoparticles can undergo plastic deformation under external forces and induce polymer matrix deformation accompanied by continuous rapid rupture and reformation of H-bonds. Therefore, fracture energy can be effectively dissipated, leading to ductility and toughness.

Employing a comprehensive methodology involving systematic mechanical experiments and MD simulations on fundamentally similar CUE_0.3_ and RC, we discovered that the water-induced viscoelastic response of hydroplastic polymers is inherently governed by their dynamic H-bond system and microstructure. The H-bonds mediated by hydroxyl groups and water molecules play a crucial role in accommodating hydrous deformation and the collective strength of the whole H-bond network. In parallel, the alterations in the material’s microstructure from anhydrous to hydrous states significantly influence its mechanical properties. This, in turn, can open the way for a universal guideline for leveraging their distinctive H-bond systems and structural characteristics. These efforts align with the current and future trend toward green chemistry and sustainable materials. They go beyond research and play a fundamental role in addressing current societal and environmental issues.

## Supporting Information Available

Experimental Section, ^1^H and ^13^C NMR spectra, elemental analysis for CUE_0.3_, FTIR spectrum, experimental stress–strain curves in a controlled environment, DMTA analysis, including the sweeps, cyclic stress–strain tests, mechano-creep and mechano-stress relaxation curves, DVS, TG and DSC curves, luminous transmittance and haze, SFE results and MD snapshots for all compounds, quantum computing for CUE and water, MD model of RC, and MD-simulated stress–strain curves, H-bond energy, polymer and H_2_O density distribution, trajectory of a single H_2_O molecule, H-bond lifetime autocorrelation function of anhydrous and hydrous RC, MD-simulated coulomb energy, and torsion angle energy of anhydrous and hydrous CUE_0.3_ (PDF)
