# Supplementary material for: Viscoelastic Response in Hydrous Polymers: The Role of Hydrogen Bonds and Microstructure
Source: Nano Lett. 2024 Mar 12;24(12):3811–8. doi: 10.1021/acs.nanolett.4c00556 (PMC10979449; doi:10.1021/acs.nanolett.4c00556)
Supplement: Supplementary file 1 — nl4c00556_si_001.pdf [file nl4c00556_si_001.pdf]

Supporting Information for

Viscoelastic Response in Hydrous Polymers: The Role of Hydrogen Bonds and Microstructure

Wenbo Chen[1], Philip Biehl[1], Caoxing Huang[1,2], Kai Zhang[1*]*

[1]Sustainable Materials and Chemistry, Department of Wood Technology and Wood-based Composites, University of Göttingen, Büsgenweg 4, Göttingen 37077, Germany. E-mail: kai.zhang@uni-goettingen.de

[2]Co-Innovation Center of Efficient Processing and Utilization of Forest Resources, College of Chemical Engineering, Nanjing Forestry University, Nanjing, Jiangsu 210037, China. E-mail: hcx@njfu.edu.cn

The document includes:

1. Supplementary Text
2. Figures and tables (Figure S1 to S19, and Table S1-S5)
3. References

Supplementary Text

1. Experimental Section

Materials

Commercial microcrystalline cellulose with a particle size of 50 µm was purchased from SERVA Electrophoresis GmbH (Heidelberg). DMAc (99.5%), DMSO (99.5%), pyridine (99%), 10-undecenoyl chloride (97%) were purchased from Sigma-Aldrich Chemie GmbH (Steinheim). Lithium chloride (LiCl) was purchased from MP Biomedicals. Methanol (99.8%) was purchased from TH. Geyer GmbH & Co. KG. Dummy CZ-Si wafer was purchased from MicroChemicals GmbH.

Synthesis of regenerated cellulose (RC)

RC was synthesized by a previously reported method with minor modification[1]. 1 g of dried microcrystalline cellulose and 40 ml of DMAc were added to a 100 ml three-necked flask with a magnetic stir bar and attached a condenser. The mixture was stirred at 130 °C for 30 min, followed by the addition of 2.8 g of LiCl at 100 °C. Under continuous stirring, the solution was allowed to cool down to room temperature overnight, leading to a clear solution. The final solution was kept in a refrigerator at 2-4 °C for further use.

Synthesis of $CUE_{0.3}$

CUE with low DS was synthesized via homogeneous acylation of cellulose by a previously reported method with minor modifications[2]. Briefly, 1 g of dried microcrystalline cellulose and 40 ml of DMAc were added to a 100 ml three-necked flask with a magnetic stir bar and attached a condenser. The mixture was stirred at 130 °C for 30 min, followed by the addition of 2.8 g of LiCl at 100 °C. Under continuous stirring, the solution was allowed to cool down to room temperature overnight, leading to a clear solution. Thereafter, the solution was heated to 60 °C, before 10-undecenoyl chloride (1.07 ml) and pyridine (0.80 ml) were added to produce CUE. The reaction was carried out by maintaining the temperature at 60 °C with stirring for 3 h. The mixture was subsequently precipitated in 200 ml of methanol. The product was collected by centrifugation, purified by repeated precipitation in methanol and dissolution in DMSO.

Preparation of RC membranes

RC membranes were made by casting the RC solution on paired smooth and flat glass plates. The thickness of the solution between the plates was set as 2 mm. Then, the casted solutions were immersed in an acetone bath for 1 h followed by washing with a stream of cold water overnight to completely remove the solvent and LiCl. The final wet RC membranes were dried at room temperature.

Preparation of $CUE_{0.3}$ membranes

$CUE_{0.3}$ membranes were made by using solvent casting technique. The wafer substrate ($55 \times 55 \times 0.7$ mm^3) was sonicated twice at 37 Hz, 60 °C in DMSO and deionized water for 3 min, respectively, followed by blow-drying with nitrogen gas. Thereafter, it was sandwiched between two Teflon blocks. The blocks were $90 \times 90 \times 15$ mm^3 in size and the upper part owns a hollow cylinder (Ø = 50 mm, h = 15 mm) in the center, as well as threads on the margin for adjusting the level and assembly fixation. 5 ml of $CUE_{0.3}$ solution at a concentration of 10 mg· ml^{-1} was transferred into the assembled molds. After drying at 80 °C and 15 mbar in a vacuum oven overnight, as-prepared $CUE_{0.3}$ membranes were obtained.

Fourier-transform infrared (FTIR) spectroscopy

FTIR spectroscopy was conducted on BRUKER ALPHA FTIR Spectrometer (Bruker, Germany) at room temperature between 400 and 4000 cm^{-1} with a resolution of 4 cm^{-1}. The samples were measured twice per 24 scans and average spectra were generated for each sample. Before the experiment for the as-prepared samples, they were vacuum dried at 105°C for 60 h and blow-dried in nitrogen gas for 3 min. Normalized vector was performed for all collected results.

Nuclear magnetic resonance (NMR) spectroscopy

$CUE_{0.3}$ was dissolved in deuterated DMSO-d6 for liquid-state ^{1}H NMR and ^{13}C NMR measurement on a Bruker DRX 500 spectrometer (Bruker, BioSpin GmbH) with a frequency of 500 MHz for ^{1}H NMR and 125 MHz for ^{13}C NMR. Totally 65 and 16000 scans were collected for ^{1}H NMR and ^{13}C NMR spectroscopy, respectively. The repetition delay was 5 s for ^{1}H NMR and 1 s for ^{13}C NMR spectroscopy.

Elemental analysis

Elemental analysis was performed on an elemental analyser Vario EL III CHN instrument from Elementar (Hanau).

Scanning electron microscopy (SEM)

The images of cross-section and surfaces of $CUE_{0.3}$ membranes were measured using an SEM Leo SUPRA 35 Instrument (Carl Zeiss SMT GmbH). A carbon layer of 10 nm was vacuum-coated on the samples before observation.

Static mechanical measurement

Mechanical tests were performed on a Z3 micro tensile test machine with a 50N load cell (Grip-Engineering Thümler GmbH). The membrane strips with a dimension of 30 × 5 mm^2 were loaded into the test machine with a clamp distance of 8.0 mm and subjected to uniaxial extension with a constant rate of 2 $mm \cdot min^{-1}$ until rupture. In each case, at least three equal samples were measured to ensure the accuracy of the results. The stress was calculated by dividing force by cross-section area, while the strain (%) was defined as $(L - L_0)/L_0 \times 100\%$, where L is the instantaneous length and L_0 is the initial length of the specimen. Toe compensation was performed before any calculation to obtain correct values by using stress-strain curves. The calculation of tensile strength, Young's modulus, elongation at break and fracture energy, as well as the toe compensation were performed according to ASTM D882-02[3]. Hydrous samples were prepared by placing the as-prepared samples in a pre-designated environment (RT, 90.0-96.9% RH) for 7 days.

Static Contact Angle and Surface Free Energy Analysis (SFE)

The surface wetting properties of the samples were assessed on a Drop Shape Analysis System (DSA 25E, Krüss) at 25°C and 60% RH. The static contact angle value of Milli-Q water and diiodomethane were measured by using the sessile droplet method with a dosing volume of 1.0 µL and a dosing rate of 1.0 µL/s. All measurements were performed at least three times, and the static contact angle was acquired by taking the mean from more than 10 equivalent measurements. Static contact angles were measured with the Young–Laplace equation and SFE was calculated with the Owens–Wendt–Rabel–Kaelble (OWRK) method.

Dynamic Mechanical Thermal Analysis (DMTA)

DMTA measurements were carried out by using a DMA GABO EPLEXOR system (NETZSCH GABO Instruments GmbH) with a force sensor of 50 N. The membrane strips with dimensions of 30 mm × 5 mm were loaded into the machine with a clamp distance of 8.0 mm. The measurements were executed with a contact force of 0.5 N, a static strain of 0.5%, and a dynamic strain of 0.1%. Humidity sweep mode was performed with RH increasing rate of 2 % $\cdot$min^{-1} at 25 °C and frequency of 10 Hz. Temperature sweep mode was performed with temperature increasing rate of 3.0 °C min^{-1} and frequency of 10 Hz. Time sweep mode was performed at 25 °C and frequency of 10 Hz. Frequency sweep mode was performed with 5 of the logarithmic intervals at 25 °C. The cyclic stress-strain measurements were performed in a length-control mode at a length rate of 2%$\cdot$ min^{-1} with a contact force of 0.1 N. There is no interval during the cyclic loading and unloading sequence.

Consecutive mechano-creep experiments were carried out at 25 °C under constant RH of 90% with a contact force of 0.5 MPa. Before testing, the membrane strips were equilibrated for 900 s, followed by the pre-programmed cyclic loading and unloading sequence. The loading stress steps were 2.5, 6.5, 10.5 and 14.5 MPa for 2 hours each. The partial unloading time steps were 300, 300, 600, and 600s. Consecutive mechano-stress relaxation experiments were measured at 25 °C under constant RH of 90% with a contact loading of 1.5 MPa. Before testing, the membrane strips were equilibrium for 900 s, followed by the pre-programmed cyclic loading and unloading sequence. The loading strain steps were 1, 3, 5, 7% for 2 hours each. The unloading time steps were 1800 s each. All measurements were carried out within the linear elastic region (**Figure S18**). For each case, three parallel tests were performed to ensure the accuracy of the collected results. Hydrous samples were prepared by placing the as-prepared samples in a pre-designated environment (RT, 90.0-96.9% RH) for 7 days.

Dynamic vapor sorption (DVS)

The dynamic water vapor sorption behavior of as-prepared samples was studied using a DVS intrinsic apparatus (DVS Advantage, Surface measurement systems). The membrane with a mass of ~9.5-10.5 mg was prepared in a dimension of 10 × 10 mm^2. The measurement was executed at 20 °C with RH increased in steps in the following pre-programmed sequence (0, 5, 10, 15, 20, 30, 40, 50, 60, 70, 80, 85, 90, and 95% RH), before decreasing to 0% RH in

the reverse order. The instrument maintained a constant target RH until the sample moisture content change (dm/dt) was less than 0.001% per minute over a 10 min period. In practice, this does not mean that a true equilibrium is reached, but previous studies have established that this allows for obtaining equilibrium moisture content (EMC) values within 0.1% of the true equilibrium value (i.e., at infinite time). The EMC at each RH was calculated on the basis of the equilibrated mass of the membranes.

Luminous transmittance and Haze

The luminous transmittance and haze of the samples were measured on the transparency Test System & Haze Meter Model of haze-gard i from BYK (Emmeram Karg Industrietechnik). The membrane surface is illuminated perpendicularly and the transmitted light is measured photo-electronically using an integrating sphere (0°/diffuse geometry). The samples were prepared in a dimension of 10 × 10 mm^2. All the measurements were tested for five times at ambient environment. The results of luminous transmittance and haze, as well as calibration were performed according to the ASTM-D1003.

Atoms in molecules (AIM) theory analysis

All-electron DFT calculations have been carried out by the latest version of ORCA quantum chemistry software (Version 5.0.3)[4]. For geometry optimization calculations, the corrected version of r2SCAN exchange-correlation functional proposed by Grimme (so-called r2SCAN-3c) was adopted[5]. The singlet point energy calculations were performed with B3LYP functional and ma-def2-TZVPP basis set[6]. The DFT-D3 dispersion correction with BJ-damping was applied to correct the weak interaction to improve the calculation accuracy[7]. Electron density analysis of bond critical point (BCP) was performed by Multiwfn software[8]. The H-bond energy was calculated using the B3LYP generalized function with DFT-D3 (BJ) dispersion correction, and the wave function was calculated by combining the ma-TZVPP basis set, while analyzing the bond critical point electron density and fitting with the following formula[9].

Molecular model of $CUE_{0.3}$ and RC

The representative molecular models of $CUE_{0.3}$ and RC were generated from initial configurations composed of 4 molecular chains with the degree of polymerization (DP) of 40,

similar to previous studies[10]. The molecular chain of $CUE_{0.3}$ was configurated in the way that the substitution site of the 10-undecenoyl group was anchored to 30% amount of the C_6 hydroxyl group in the RC molecular chain. The thermal results of TG and DSC exemplified the amorphous nature of $CUE_{0.3}$ and RC membranes (Figure S19). The amorphization processing of the molecular models was conducted as follows: the molecular chains were packed parallelly into a large cubic box with the edge length of 200 Å, ensuring large spacing between these molecular chains. By such arrangement, they are able to freely twist and fold, thereby leading to the loss of long-range ordering and formation of amorphous isotropic phase[10]. The density of resulting amorphous $CUE_{0.3}$ and RC is 1.38 and 1.58 $g{\cdot}cm^{-3}$, respectively.

Prior to Molecular Dynamics (MD) simulation, these initial configurations were quasi-statically relaxed to a local minimum-energy configuration through conjugate gradient method, in which the energy and force tolerances were set to be 1.0×10^{-4} Kcal/mol and 1.0×10^{-4} Kcal/(mol·Å), respectively. Afterwards, as-minimized configurations were relaxed by MD simulations with 20 ps at 10 K and zero confining pressure, and then further relaxed via increasing temperature from 10-700 K within 100 ps. Then, MD simulations with 2 ns were performed at temperature of 700 K and zero pressure to obtain amorphized structures. Subsequently, the amorphized cellulose-based structures were quenched to the temperature of 300 K and equilibrated for 10 ns under both NVT and NPT ensembles[11]. The temperature and pressure are controlled using Nosé–Hoover thermostat and barostat method with damping times of 100 and 1000 timesteps. Periodic boundary conditions (PBCs) were applied in the systems. As for the hydrous $CUE_{0.3}$ and RC models, similar MD procedure was conducted excepted for inserting desired number of water molecules. The system is characterized by a water content value:

$$\mathrm{m} = \frac{N_{H_2O} \times M_{H_2O}}{M_{polymer}^{dry}} \tag{1}$$

where N_{H_2O} is the number of water molecules in the system, M_{H_2O} is the mass of a water molecule, and $M_{polymer}^{dry}$ is the mass of the polymer in dry state. The maximum moisture content investigated is taken from DVS experiment. The MD time step was assigned to be 1.0 fs by the velocity-Verlet algorithm to integrate the Newton's motion.

Molecular dynamics (MD) simulations

The molecular dynamics simulations were carried out using the ReaxFF reactive force field as implemented in the Large-scale Atomic/Molecular Massively Parallel Simulator (LAMMPS) simulation package[11-12]. This force field has been shown to provide an accurate description of hydrocarbons and water[11]. ReaxFF also allows for an explicit consideration of long-range and nonbonded interactions such as H-bonds and coulombic interactions making it particularly suitable for predicting reasonable structural and mechanical properties for materials dominated by H-bonds.

Prior to the mechanical testing, as-obtained amorphized configurations were further relaxed by MD simulations with first-principles-based ReaxFF[11] for 100 ps at 300 K and zero confining pressure under NPT ensemble, and followed by 100 ps at 300 K under NVT ensemble. Finally, uniaxial stretching with engineering straining rate of 0.000001/fs was applied by increasing the length of one (x) direction of simulation box at 300 K. During the uniaxial stretching, zero confining pressure was maintained at the lateral directions to introduce the Poisson effect. Atomic stresses in the investigated systems were computed on the basis of the definition of virial stress. The timestep being 0.25 fs. ReaxFF forcefield developed by Strachan et al.[13] was employed to examine the mechanical properties of our specimens. All the MD calculations were implemented using the LAMMPS package code. In addition, the Polymer Consistent Force Field (PCFF)[14] was employed for the molecular dynamics simulations, in order to describe the lifetimes of the H-bonds in the systems.

The dynamic behavior of the H-bond was calculated via the time autocorrelation function of the presence of H-bonds[15]:

$$\mathrm{C}(\tau) = < \frac{h_{ij}(t_0)\, h_{ij}(t_0 + \tau)}{h_{ij}(t_0)^2} > \quad (2)$$

where h_{ij} indicates the presence of a H-bond between atoms i and j:

h_{ij}= 1 if there is a H-bond

h_{ij}= 0 if there is no H-bond

$h_{ij}(t_0)$= 1 indicates there is a H-bond between atoms i and j at a time origin t_0, and $h_{ij}(t_0 + \tau)$= 1 indicates these atoms remain hydrogen bonded throughout the period t_0 to $t_0 + \tau$. To improve statistics, multiple time origins, t_0, are used in the calculation and the average is taken over all time origins.

Two distinct types of autocorrelation functions, continuous and intermittent are computed in this study. The continuous time autocorrelation function assesses the lifespan of the H-bonds, considering it continuously bonded from t_0 to t, without accounting for the reformation of the same bond, even if it occurs at a later time. In contrast, the intermittent correlation function provides the probability of a specific tagged H-bonds remaining intact at time t given that it was intact at the initial time t_0. Consequently, C(t) is unaffected by the breaking of H-bonds at any intermediate time and allows for the reformation of broken bonds.

2. Nuclear magnetic resonance (NMR) analysis

Signals from both cellulose backbone and undecenoyl groups were observed from liquid-state ^1H and ^{13}C NMR spectra. Within the ^1H NMR spectrum (Figure S1A), the signals around 4.9 and 5.8 ppm represent protons of terminal olefin protons of 10-undecenoyl groups. The peaks between 2.9 to 5.2 ppm originate from cellulose backbone except for the signal at 4.9 ppm. The peaks between 1.2 and 2.3 ppm are derived from the protons in saturated aliphatic chains of 10-undecenoyl moieties[16]. Within the ^{13}C NMR spectrum (Figure S1B), the peak at 173 ppm was attributed to the carbons in C=O groups at the C_6 position of the anhydroglucose units (AGUs) of cellulose[17]. The peaks at 114 and 139 ppm denote the carbons in terminal olefin groups, while the weak signals from 50 to 105 ppm were derived from cellulose backbone. The signals from 10 to 40 ppm correspond to the saturated aliphatic chains of 10-undecenoyl moieties. Moreover, C_6 of the AGUs of cellulose has a new signal at ~63 ppm in addition to the signal at ~60 ppm, showing the esterification at C_6 position. In comparison, the signal attributed to C_1 at ~103 ppm is not split, indicating no modification of C_2 position.

3. Fourier-transform infrared (FTIR) spectroscopy

Compared to the FTIR spectrum of RC, the bands related to vinyl groups at 3077, 1740, 1640 cm^{-1} emerged, which are attributed to stretching vibrations of CH_2=CH_2, stretching vibrations of C=O, stretching vibrations of C=C, respectively[16, 18]. The intensities of signals at 2927 and 2855 cm^{-1} strongly increased, which is due to the asymmetric and symmetric

stretching vibrations of C-H groups in alkyl chains, respectively[18]. The above new signals represent the successful synthesis of $CUE_{0.3.}$

4. Elemental analysis

The degree of substitution (DS) of CUE was calculated according to previously published method based on the content of carbon with modification[19]:

$$\mathrm{DS} = \frac{162.14 \times \mathrm{c} - 72.066}{132.121 - 166.264 \times c} \tag{3}$$

Where C% is the content of elemental carbon and n is the number of carbon atoms in the acyl moieties, respectively. The carbon content of CUE was measured to be averagely 51.5% using elemental analysis.

5. Static mechanical properties

Static mechanical properties of as-prepared $CUE_{0.3}$ and RC membranes at dry state as well as those after equilibrium hydration at 90% RH for 7 days were analyzed **(Figure S12)**. Compared to the mechanical properties of as-prepared $CUE_{0.3}$ membranes, their Young's modulus at hydrous state only showed minor change from 1.3 ± 0.1 to 1.2 ± 0.1 GPa, while their tensile strength decreased from 72.8 ± 1.9 to 59.3 ± 2.5 MPa, the elongation at break increased from 17.4 ± 2.4 to 26.4 ± 3.4%, and the fracture energy increased from 9.9 ± 1.3 to 11.8 ± 1.1 MJ/m^3. In addition, compared to the mechanical properties of anhydrous RC membranes, their Young's modulus in equilibrium hydrous state showed dramatic decline from 1.5 ± 0.1 to 0.12 ± 0.01 GPa, while the tensile strength decreased from 170.1 ± 5.9 to 59.3 ± 3.9 MPa, the elongation at break showed minor change from 49.7 ± 3.2 to 50.8 ± 3.3%, and the fracture energy decreased from 62.1 ± 7.9 to 13.7 ± 1.2 MJ/m^3. Despite the different molecular compositions, the $CUE_{0.3}$ and RC membranes in hydrous state exhibited linear stress-strain responsiveness at low strain of 1%. Surprisingly, they demonstrated similar stress-strain responsiveness as tensile deformation was 5% and higher, despite their varying strengths at equivalent strain level. These results can be primarily attributed to the comparable molecular interactions among the hydrous $CUE_{0.3}$ polymer chains and the hydrous RC polymer chains[20].

6. Supplementary text on the Dynamic Mechanical Thermal Analysis (DMTA)

As-prepared materials were measured in 10% RH representing their intrinsic mechanical properties in anhydrous state. In parallel, the hydrous materials were measured in an environment with 90% RH, representing their mechanical properties in water-equilibrium state.

The anhydrous $CUE_{0.3}$ membranes showed storage modulus, loss modulus and damping factors of 3.10 ± 0.3 GPa, 0.20 ± 0.04 GPa and 0.065 ± 0.005, respectively. The hydrous $CUE_{0.3}$ membranes showed storage modulus, loss modulus and damping factors of 0.96 ± 0.08 GPa, 0.14 ± 0.01 GPa and 0.137 ± 0.001, respectively.

The anhydrous RC membranes showed storage modulus, loss modulus and damping factors of 6.5 ± 0.2 GPa, 0.24 ± 0.03 GPa and 0.038 ± 0.005, respectively. The hydrous $CUE_{0.3}$ membranes showed storage modulus, loss modulus and damping factors of 1.51 ± 0.18 GPa, 0.24 ± 0.01 GPa, and 0.142 ± 0.004, respectively.

At a temperature of -80 °C, the hydrous $CUE_{0.3}$ membranes depicted storage and loss modulus of 5.9 ± 0.1 GPa and 0.49 ± 0.01 GPa, while hydrous RC membranes showed storage and loss modulus of 10.7 ± 0.1 GPa and 0.75 ± 0.02 GPa, respectively.

The results of humidity sweep were shown as follows. The storage modulus of as-prepared $CUE_{0.3}$ membranes decreased from 3.19 ± 0.29 GPa to 1.34 ± 0.07 GPa in the RH range of 18 to 90%. Correspondingly, the damping factors rose from 0.053 ± 0.002 to 0.133 ± 0.003. In comparison, the storage modulus of as-prepared RC membranes decreased from 6.46 ± 0.26 GPa to 1.97 ± 0.09 GPa. Correspondingly, the damping factors rose from 0.047 ± 0.005 to 0.143 ± 0.006.

Moreover, the results of consecutive plasticity mechano-creep experiments of hydrous $CUE_{0.3}$ and RC membranes are shown **in Table S4**. Stress relaxation measurements can be useful in investigating the molecular-level processes of relaxation under load. Since the sample is kept under a constant deformation due to the imposed step strain, molecular-level movement rather than macroscopic change dominates the stress relaxation processes. As shown in **Figure S5d**, hydrous RC membranes exhibit a weaker capacity for retaining the residual stress compared to the hydrous $CUE_{0.3}$ membranes. Therefore, an excessive water-induced plasticization may lead to premature material failure.

The mathematical results of consecutive plasticity mechano-stress relaxation experiments on hydrous $CUE_{0.3}$ and RC membranes are shown **in Table S5**. The residual stress was calculated by the following equation:

$$\text{Residual stress } (\%) = \frac{\sigma_e}{\sigma_0} \quad (4)$$

Where σ_e , σ_0 represent the equilibrium and initial stress in each strain cycle, respectively.

In addition, the storage modulus of as-prepared $CUE_{0.3}$ and RC membranes dropped by 58% and 69.5% with increasing RH from 18 to 90%, respectively. Correspondingly, the damping factors increased by 2.5 and 3.0 folds, respectively **(Figure S6a-d)**. Compared to the anhydrous state, the storage modulus and damping factors of hydrous $CUE_{0.3}$ and RC membranes exhibited dramatic change, but remained stable after 4 h at 10 Hz **(Figure S7a-d)**. These results exemplify the prominent impact of water on the mechanical properties of $CUE_{0.3}$ and RC membranes. Notably, the storage and loss modulus of hydrous $CUE_{0.3}$ membranes were only 63.6% and 58.3% of those observed in hydrous RC membranes, respectively, yet similar value of damping factors **(Figure S7e-g)**. Moreover, time sweep experiments on hydrous $CUE_{0.3}$ and RC membranes showed their robust structural integrity and long-term mechanical durability even at extremely low temperatures, such as -80°C **(Figure S7a-d, S8)**.

<u>7. Water-Induced Transition of Structure and Hydrogen Bonds System in RC Membranes</u>

RC exhibits a notably distinct polymer density distribution compared to $CUE_{0.3}$ **(Figure S9a)**. The large interconnected low-density regions suggest the existence of numerous intricate pores within the system. This could be explained by the fact that more water accessible area was observed in the water density distribution of RC model, compared to the $CUE_{0.3}$ model **(Figure S9b)**. In addition, it was observed that a single water molecule could access most of the system within 20 ns **(Figure S9c)**. Water molecules should undergo changes to remain in one position due to the relatively larger pore size, which can accommodate water molecules that are not tightly bonded to the RC chains, such as multilayer water or even free water. As shown in **Figure S9d,** the decay degree of continuous

H-bond lifetime function in hydrous RC was similar to anhydrous $CUE_{0.3}$. In parallel, the decay rate of intermittent H-bonds in anhydrous and hydrous RC rapidly approaches zero and maintains a constant intermittent C(t) over an extended time scale. The intermittent C(t) value of hydrous RC at 1490 ps was half that of anhydrous RC. Furthermore, the intermolecular H-bond lifetime function in hydrous RC confirms that this unfavorable variation was attributed to the fact that water facilitates the rapid dissociation of H-bonds, exceeding the rate at which new H-bonds are formed. This phenomenon arises from the limited capability of the RC chains to capture these part of water molecules, consequently hindering the formation of mechanically stabilized RC-H_2O H-bonds. This disparity in the ability to form such bonds may constitute the primary reason for the distinct mechanical responses observed in RC and $CUE_{0.3}$ upon stretching.

8. MD simulation of the Mechanical properties in Hydrous RC Membranes

As shown in **Figure S14a**, hydrous RC exhibited mechanical characteristics of lower strength, and lower Young's modulus compared to anhydrous RC. This result agrees with findings from corresponding tensile experiments. Water molecules primarily function to increase the spacing between RC chains. Within the 20% tensile strain as in the MD simulation on mechanical tension of hydrous RC, no obvious water strengthening effect was observed. Therefore, the primary role of water molecules in RC was to increase the spacing between polymer chains. **Figure S14b-c** plots the variation of H-bond energy in anhydrous and hydrous RC as a function of 20% tensile strain, respectively. In comparison to anhydrous RC, the H-bond energy of hydrous RC decreases during the early strain stage, increases and then continuous decreases at ever larger strains. Conversely, the H-bond energy of anhydrous RC is stabilized after the initial reduction. This suggests that the mechanical response of hydrous RC is governed by RC-RC H-bonds, but water molecules can significantly weaken these bonds.

As shown in Figure S15, no strain hardening behavior was observed before 20% tensile strain in the MD simulation on mechanical tension of anhydrous RC model. This finding contrasts with the strain-hardening behavior observed in $CUE_{0.3}$ due to the alignment of polymer chains. These results indicate that brittle failure of RC in anhydrous or hydrous states was due to the breakage of RC-RC H-bonds.

9. Dynamic vapor sorption (DVS) analysis

DVS analysis was executed to reveal behaviors of $CUE_{0.3}$ and RC membranes at varied RH and the number of available interaction sites for water vapor **(Figure S10a)**. The sorption behavior was analysed with the Guggenheim–Anderson–de Boer (GAB) model[21], which is usually expressed as:

$$M = \frac{CKM_0 a_w}{(1 - Ka_w)(1 - Ka_w + CKa_w)} \tag{5}$$

Where M is the equilibrated water content (g per 100 g solid), a_w is water activity (RH), M_0 is the water content in a monolayer which indicates the active -OH sites on $CUE_{0.3}$ and RC that are available for water, C is the Guggenheimer constant which represents the strength of water-binding primarily to the $CUE_{0.3}$ and RC polymer chains, and K is a constant related to the different sorption heat of multilayer water and bulk liquid water. The fitted R^2 value by adopting the GAB model reaches 0.999, which indicates a well fit of the model with the sorption curve of $CUE_{0.3}$ and RC membranes. In parallel, the M, M_0, k and C values were 0.15, 4.0, 0.8 and 5.4, as well as 0.30, 6.4, 0.8 and 6.9 for $CUE_{0.3}$ and RC membranes, respectively. These results are based on the fact that RC membranes possess more -OH groups. They could act as the water adsorption sites (larger M_0), and in turn result in the accumulation of more multilayer water within the interior. The larger hysteresis loop area of RC than that of $CUE_{0.3}$ is resulted from the response delay caused by the collapse of nanopores in the matrix as more internal water molecules exit as well as the delay of structural deformation during the adsorption process **(Figure S10b)**. The equilibrium time of $CUE_{0.3}$ and RC membranes during the sorption and desorption cycle was evaluated by normalizing the time by dividing the actual time by the mass of the corresponding sample. RC membranes exhibited slower sorption and desorption speed than that of $CUE_{0.3}$ membranes **(Figure S10c)**.

In addition, the cluster number of water vapor in dependence on water activity was estimated based on GAB fitting according to the following equation:

$$N_c = -(1 - \varphi_1) \times \left(\frac{\varphi_1}{M_0 C}(-2CKa_w + 2Ka_w + C - 2) - 1\right) \tag{6}$$

Where N_C is the cluster number of water and φ_1 is the volume fraction of water. No substantial water cluster was observed in $CUE_{0.3}$ and RC membranes. This implies the monolayer sorption of water vapor molecules was closely associated with the accessibility of -OH sites. Several reasons could account for this result. On the one hand, water vapor was

more inclined to bind new -OH sites rather than aggregate into clusters due to the inherent strong polarity of $CUE_{0.3}$ and RC polymer chains. On the other hand, the sorption of water vapor was impeded due to the amphiphilic nature and the high energy barrier of polymer chains, hindering the diffusion of water vapor to a certain depth. This statement was consistent with the static water contact angle of $CUE_{0.3}$ membranes (88.07 ± 1.29^{o}) and RC membranes (86.43 ± 2.48^{o}).

10. Surface free energy (SFE) analysis

Wetting ability analysis was conducted to elucidate the surface free energy properties of these membranes. The relative proportion between the polar and dispersive components in SFE will help to reflect the competition between hydrophilicity and lipophilicity which governs the wettability of the solid surface.

The disperse-dominated SFE affords the surfaces of $CUE_{0.3}$ membranes more affinity to resist large number of water molecules, showing a stability of the bulk materials even under atmosphere of continuous high humidity level. Moreover, the ratio of polar SFE to disperse SFE, denoted as p/d, can accurately reflect the competitive relationship between hydrophilicity and lipophilicity of a solid surface. The higher p/d value, the stronger interfacial affinity to water. The p/d values were calculated from **Table S1** as 9.17% for $CUE_{0.3}$ membranes and 15.92% for RC membranes. This could explain why RC membranes showed a stronger interfacial hydrophilicity.

11. Optical properties

As shown in **Table S1**, both of the $CUE_{0.3}$ and RC membranes are flat, highly transparent with low haze, which represent their homogeneous microstructure.

12. Quantum computing of H-bond energy between CUE and water

Optimized atomic configuration of minimum point on the potential energy surface of CUE-H_2O showed the H-bond pattern and binding sites between $CUE_{0.3}$ polymer chains and H_2O, which is expected to be consistent with the conformational changes that take place

within $CUE_{0.3}$ matrix (Figure S14). The H-bond energy was calculated using the B3LYP generalized function with DFT-D3 (BJ) dispersion correction, and the wave function was calculated by combining the ma-TZVPP basis set. The bond critical point electron density was then calculated and fitted with the following formula[9]:

$$\Delta E \approx -223.08 \times \rho(r_{BCP}) + 0.7429 \tag{7}$$

The calculated results of electron density and H-bond energy between $CUE_{0.3}$ and water are shown in **Table S3**.

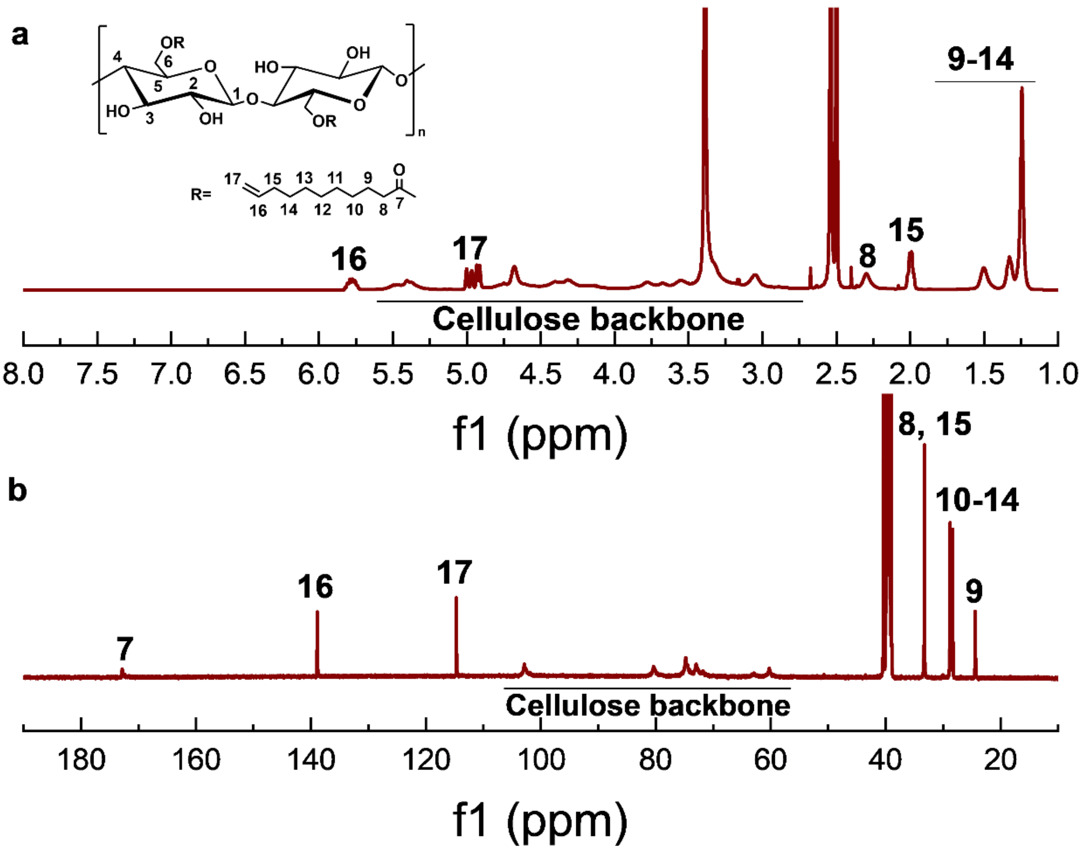

Figure S1.

(a) ^{1}H and **(b)** ^{13}C NMR spectra of cellulose 10-undecenoyl esters ($CUE_{0.3}$) in DMSO-d6.

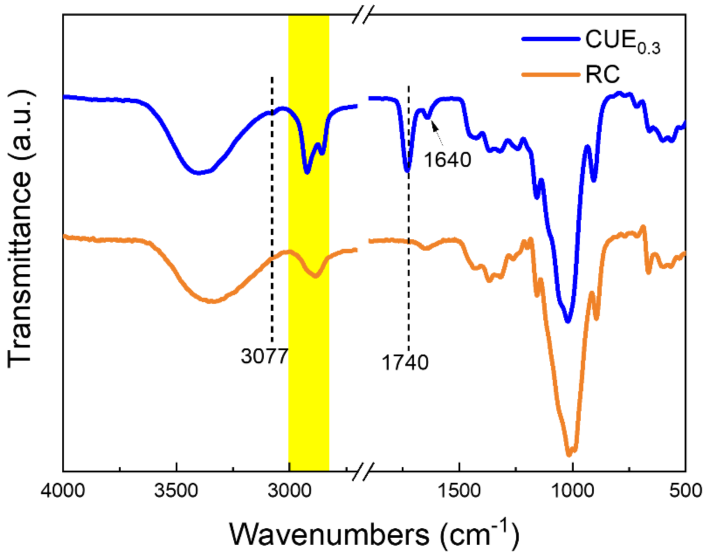

Figure S2.

FTIR spectrum of $CUE_{0.3}$ and RC.

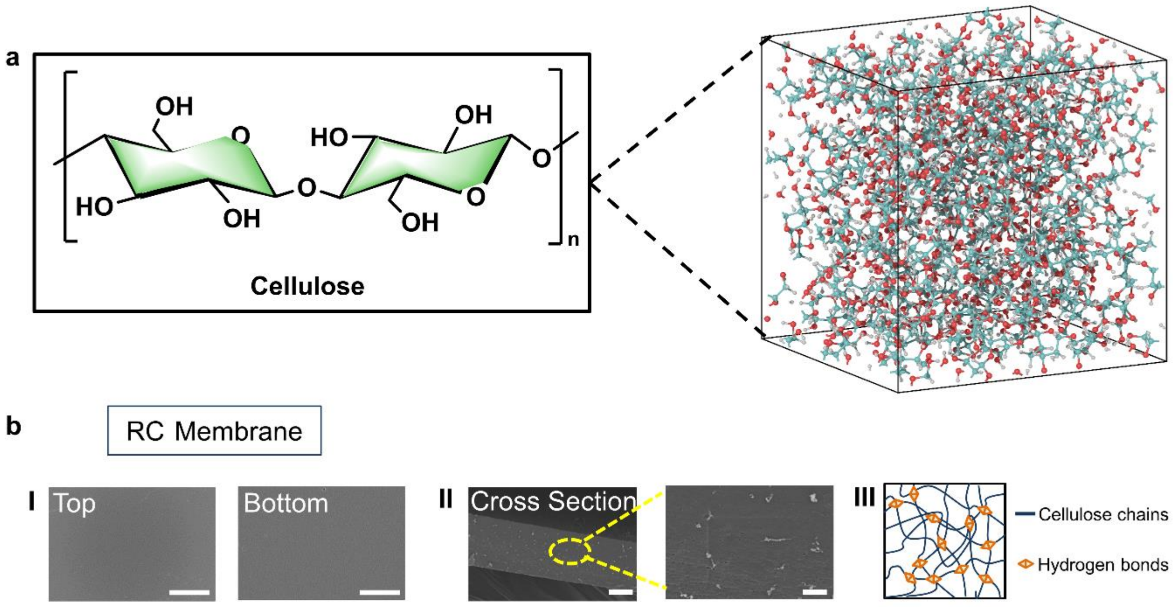

Figure S3.

a) Chemical structure of cellulose and the molecular-scale model for RC. **b)** Multiscale structure of the fabricated RC polymers. Scanning electron microscopy image in **(i)** the top and bottom surface, **(ii)** the cross-section and enlarged cross-section of the hydrous RC membranes. **(iii)** the aggregated polymer domains with various interactions. Scale bar in **(i)** to **(ii)** is 5 µm, 5 µm, 2 µm and 300 nm, respectively.

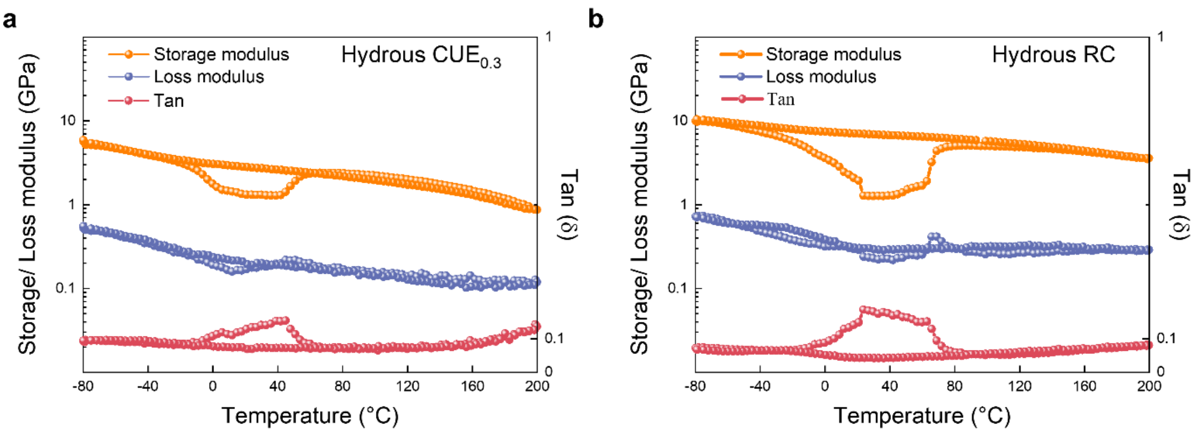

Figure S4.

Temperature sweep of hydrous **(a)** $CUE_{0.3}$ and **(b)** RC membranes from -80 to 200 °C at 10 Hz.

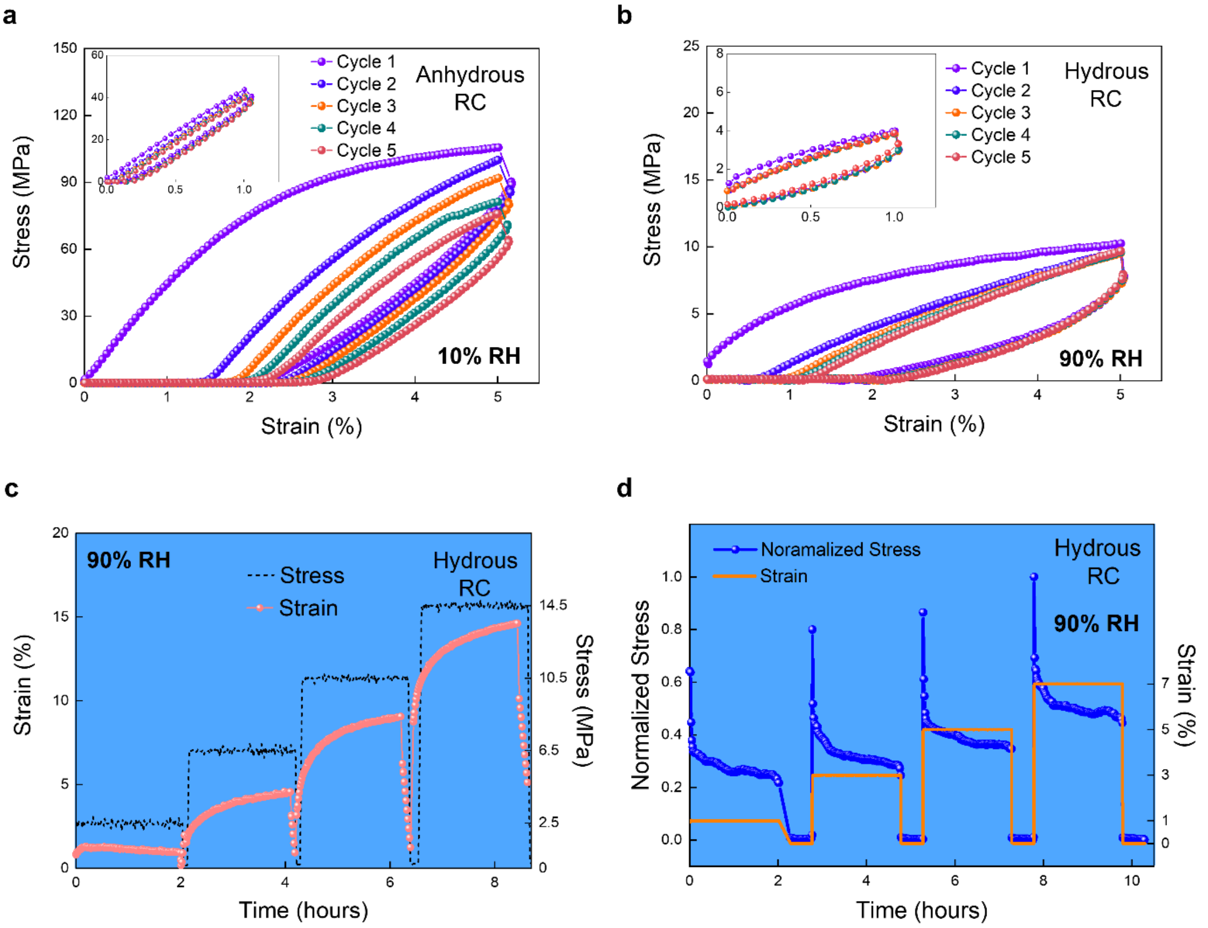

Figure S5.

Experimental cyclic stress-strain responses of **(a)** anhydrous RC membranes to 5% strain at 10% RH, as well as for **(b)** hydrous RC membranes to 5% strain at 90% RH. Insets show cyclic stress-strain responses of the same membranes to 1% strain. **c)** Consecutive plasticity mechano-creep experiments of hydrous RC membranes upon step loading and partial unloading cycles at constant 90% RH. **d)** Consecutive plasticity mechano-stress relaxation experiments of hydrous RC membranes upon loading and unloading cycles at constant 90% RH.

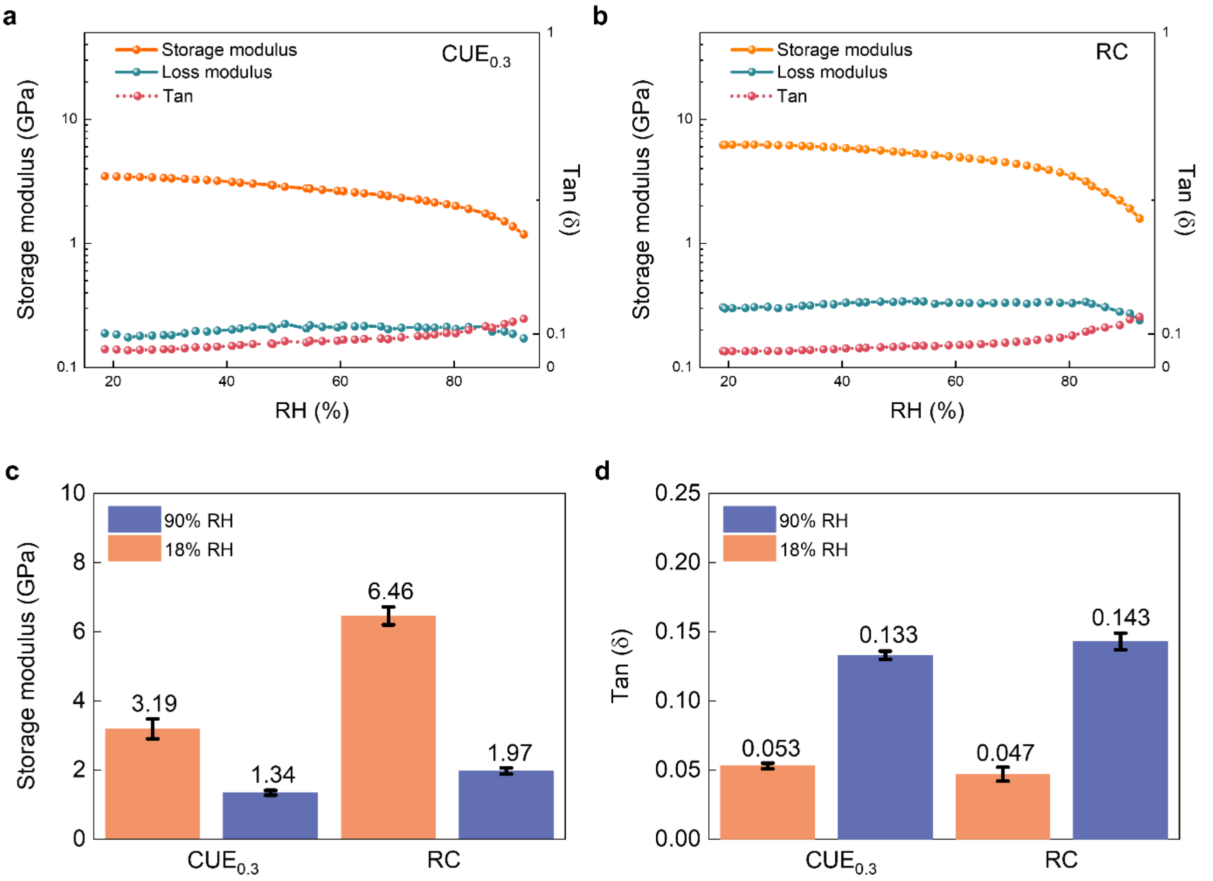

Figure S6.

Humidity sweep of as-prepared **(a)** $CUE_{0.3}$, **(b)** RC membranes from 18% to 90% RH at 10 Hz. Results as **(c)** storage modulus, **(d)** damping factors at 18% and 90% RH, respectively.

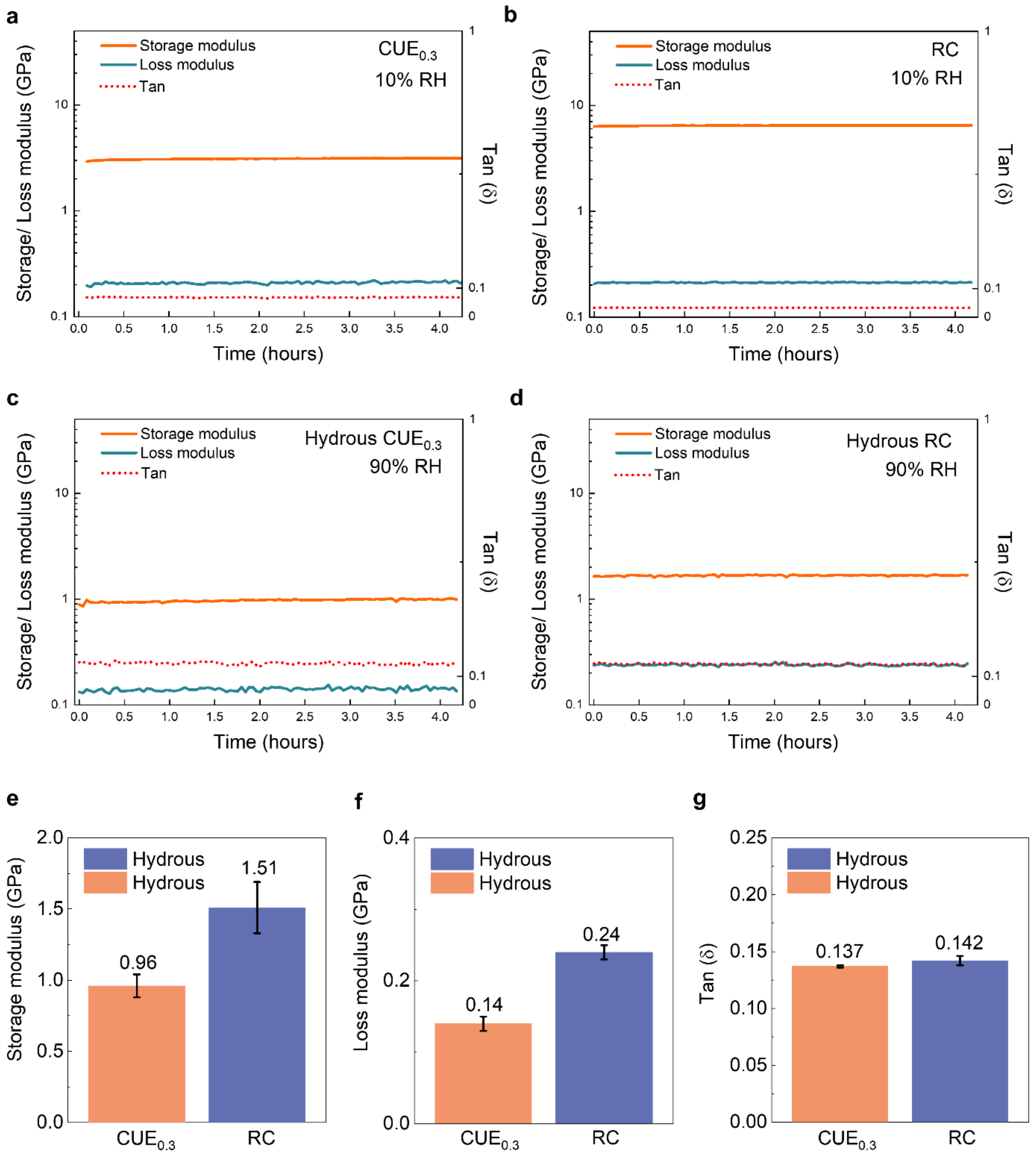

Figure S7.

Time sweep of as-prepared **(a)** $CUE_{0.3}$ and **(b)** RC membranes at 10% RH, 10 Hz. Time sweep of hydrous **(c)** $CUE_{0.3}$ and **(d)** RC membranes at 90% RH, 10 Hz. The results as **(e)** storage modulus, **(f)** loss modulus, **(g)** damping factors at 18% and 90% RH, respectively.

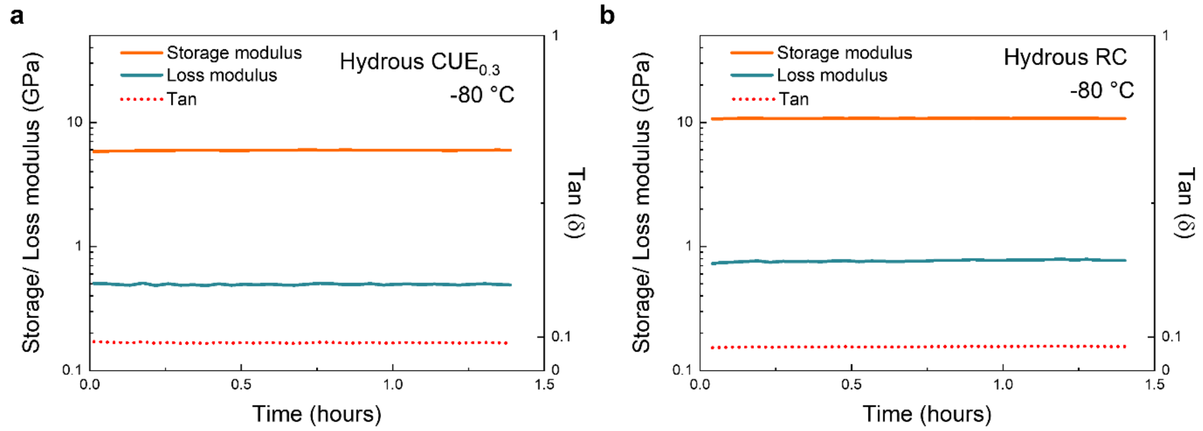

Figure S8.

Time sweep of hydrous **(a)** $CUE_{0.3}$ and **(b)** RC membranes at -80 °C, 10 Hz.

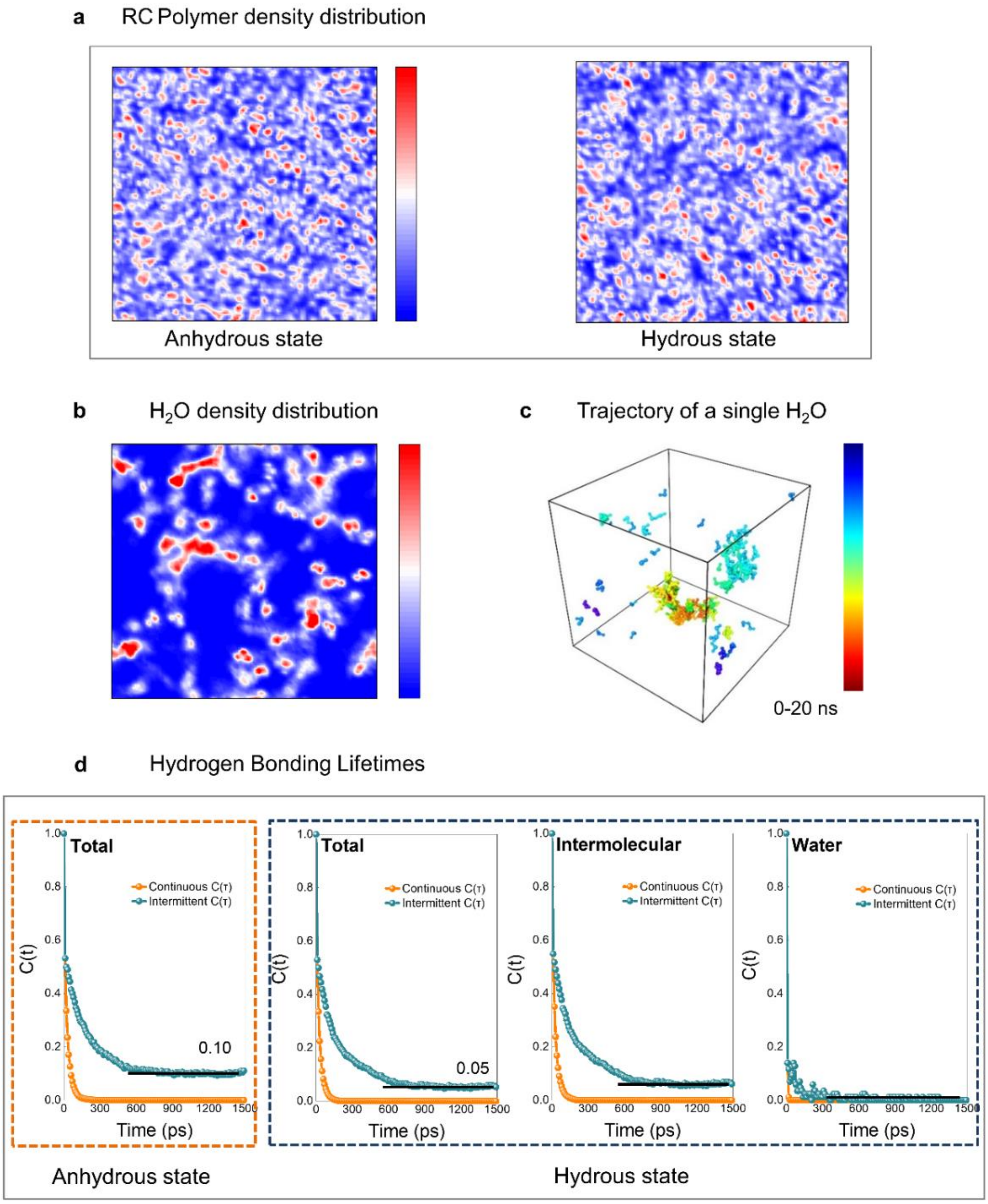

Figure S9.

a) The averaged RC polymer density distribution profile at equilibrium anhydrous and hydrous states in the MD box. **b)** The averaged H_2O density distribution profile at equilibrium hydrous state in the MD box. The red color denotes regions of space most likely to be occupied, while the blue space is not occupied. **c)** The trajectory of a single H_2O molecule during 20 ns in the MD box at equilibrium. The colors from red to blue mark the evolution of time. **d)** The continuous and intermittent hydrogen bonding lifetimes autocorrelation function of RC polymer at anhydrous and hydrous states, respectively.

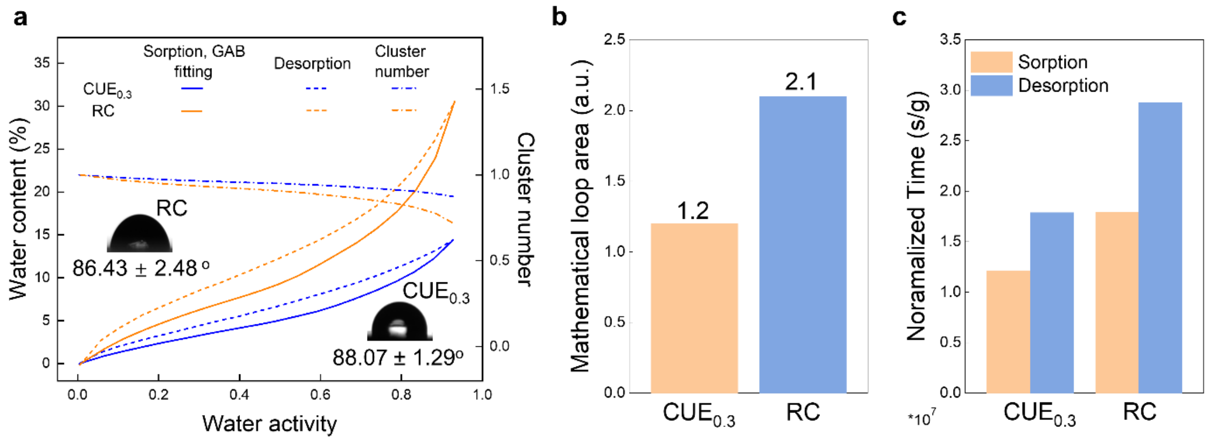

Figure S10.

a) Dynamic water vapor sorption isotherm of as-prepared $CUE_{0.3}$ and RC membranes, GAB fitting of the sorption curve and calculated water cluster number. Inset shows the static water contact angle on their surface. **b)** Mathematical loop area of as-prepared $CUE_{0.3}$ and RC membranes. **c)** Normalized time of as-prepared $CUE_{0.3}$ and RC membranes during sorption and desorption process.

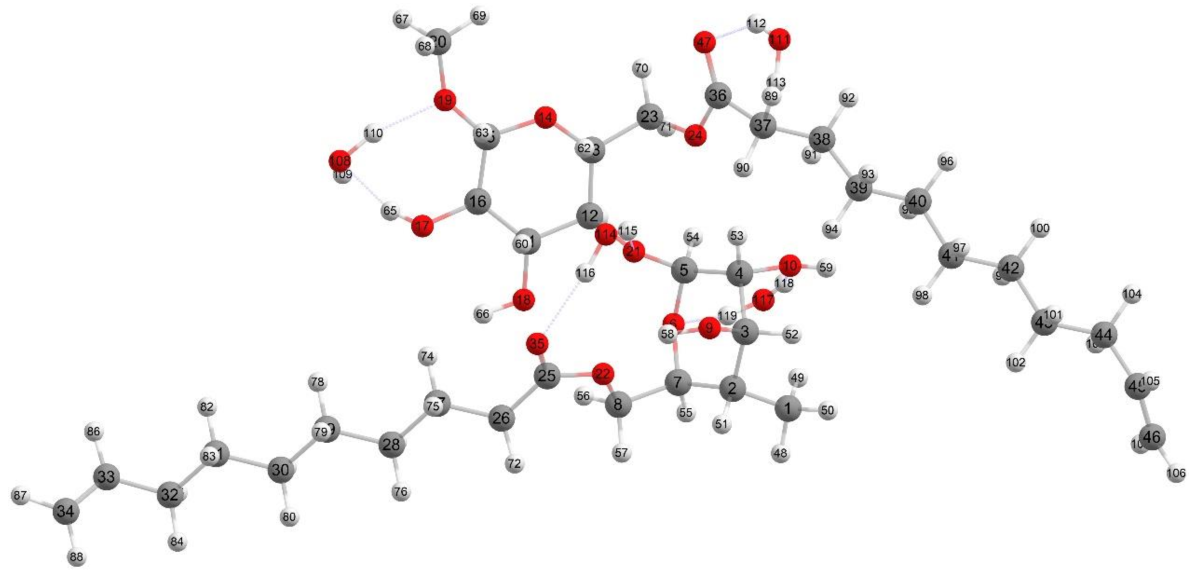

Figure S11.

Optimized atomic configuration of minimum point on the potential energy surface of CUE-H_2O based on atoms in molecules theory, showing the hydrogen bonds pattern and binding sites between $CUE_{0.3}$ and H_2O.

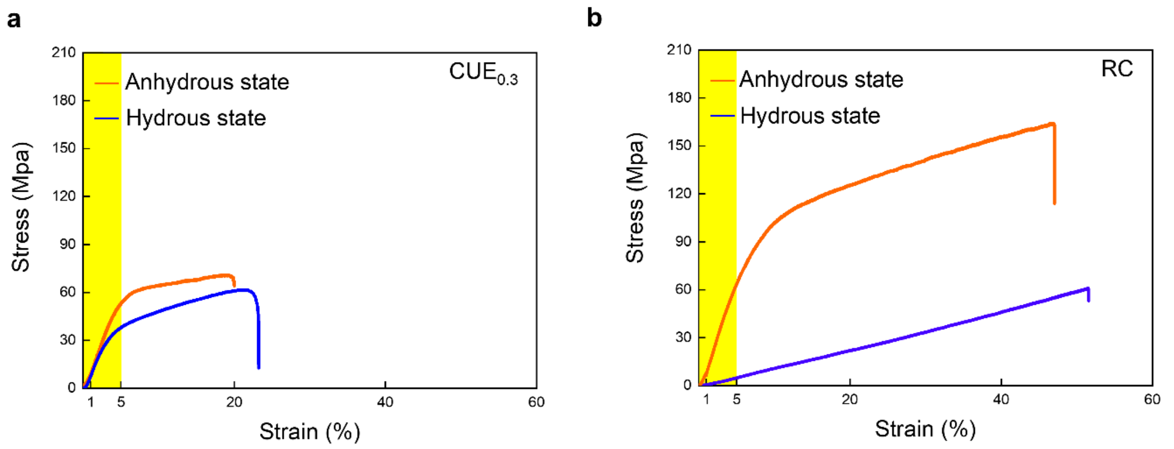

Figure S12.

Stress-strain curves of **(a)** $CUE_{0.3}$ and **(b)** RC membranes at as-prepared and hydrous states, respectively.

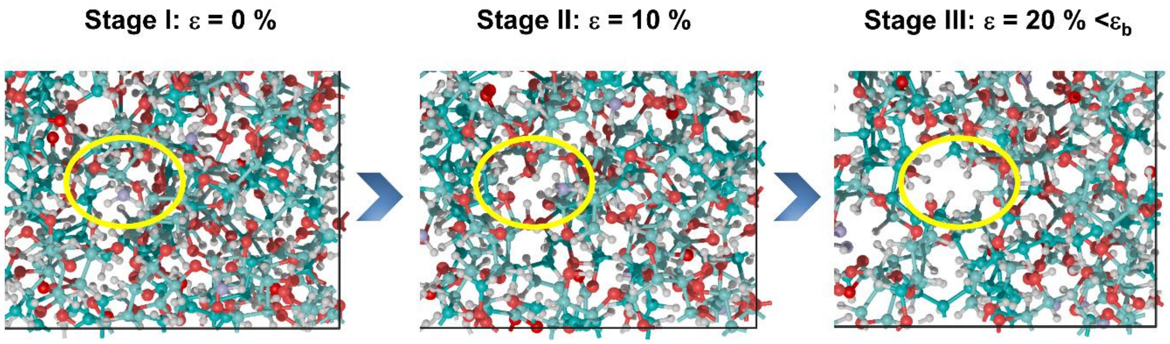

Figure S13.

Enlarged local snapshots for the MD simulated movements of hydrous $CUE_{0.3}$ polymer chains with larger strains.

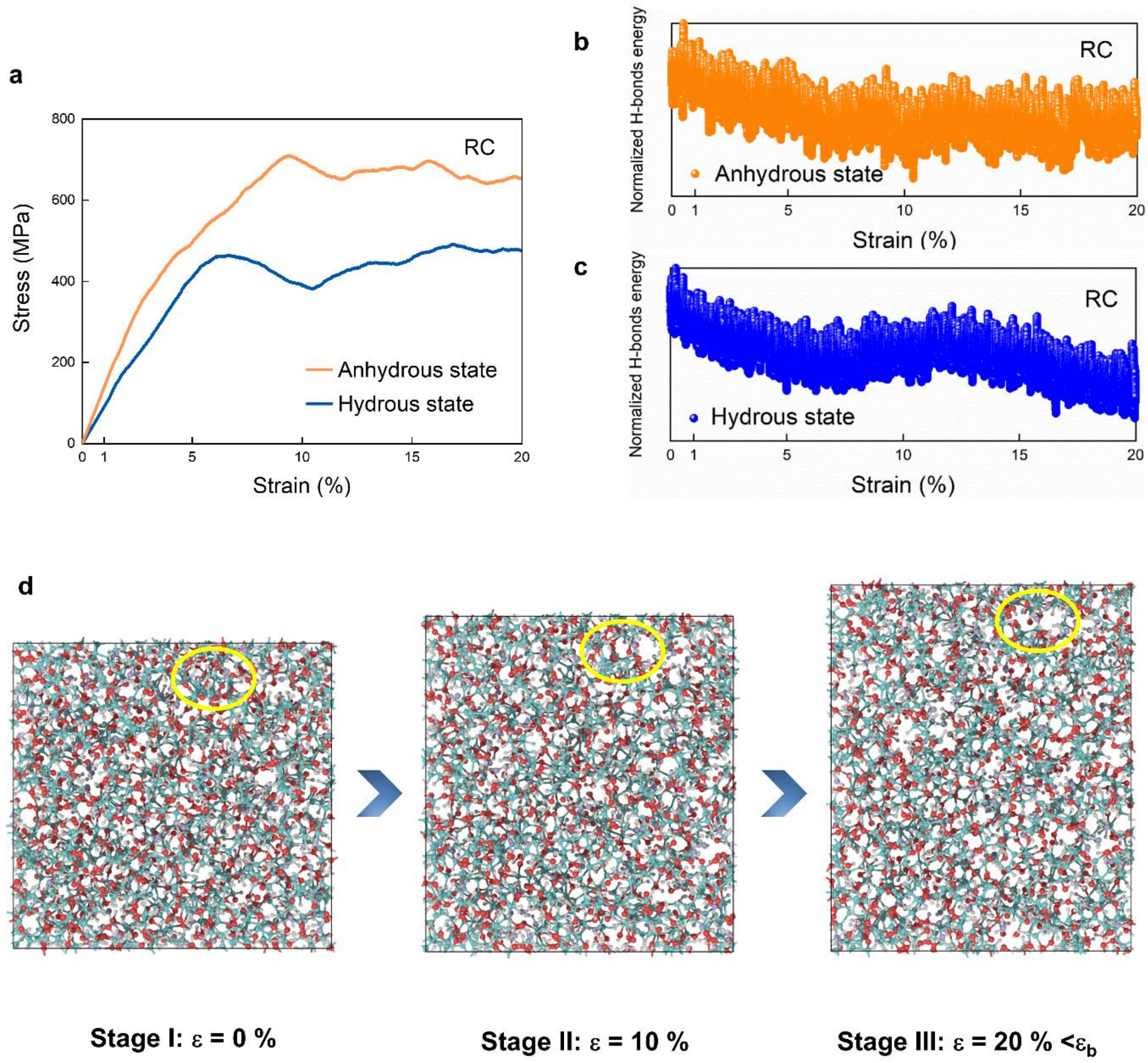

Figure S14.

a) MD simulated stress-strain curves of RC at anhydrous and hydrous states, respectively. Change of normalized H-bond energy in **(b)** anhydrous and **(c)** hydrous RC as a function of strains. **d)** Snapshots for the MD simulated movements of hydrous RC polymer chains with larger strains.

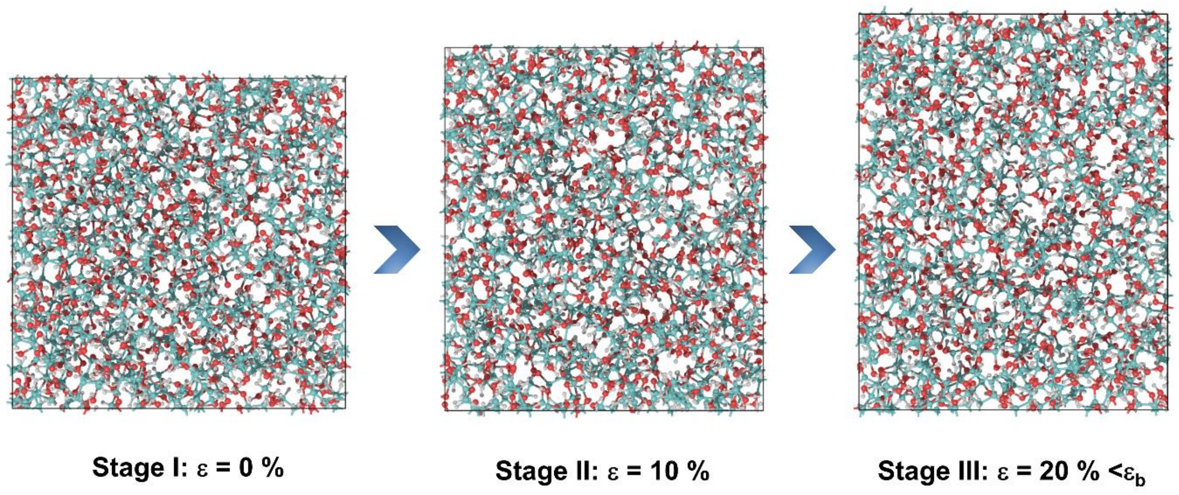

Figure S15.

Snapshots for the MD simulated movements of anhydrous RC polymer chains with increasing strains.

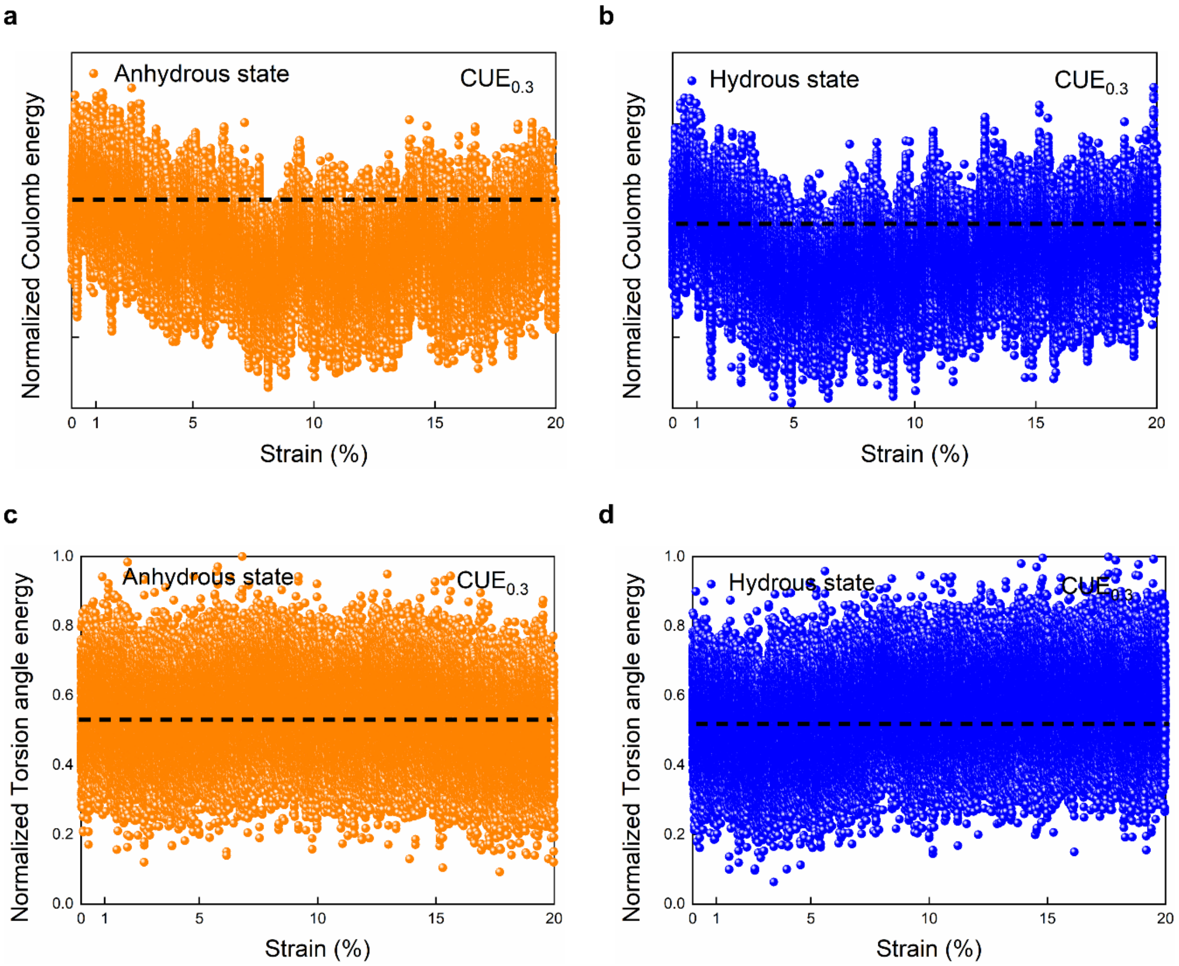

Figure S16.

Change of normalized coulomb energy in **(a)** anhydrous and **(b)** hydrous $CUE_{0.3}$ as a function of strains. Change of normalized torsion angle energy in **(c)** anhydrous and **(d)** hydrous $CUE_{0.3}$ as a function of strains.

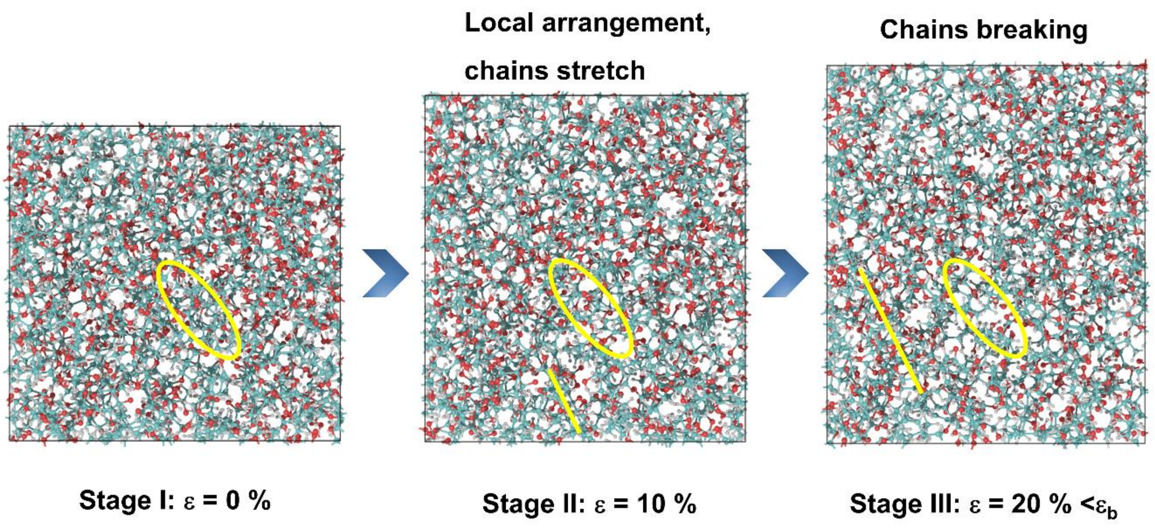

Figure S17.

Snapshots for the MD simulated movements of anhydrous $CUE_{0.3}$ polymer chains with larger strains.

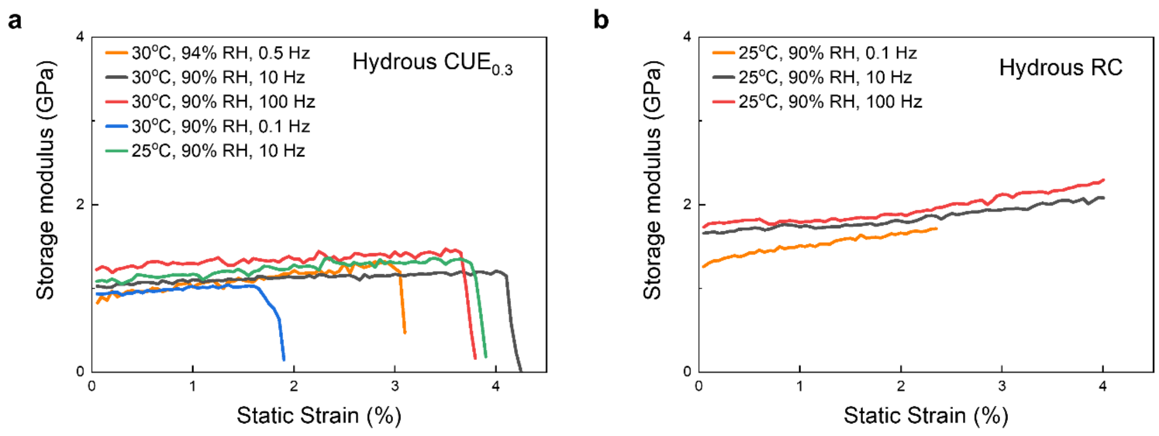

Figure S18.

Strain sweep of hydrous **(a)** $CUE_{0.3}$, **(b)** RC membranes under high RH conditions at different frequencies, representing the conditions used for DMTA measurements.

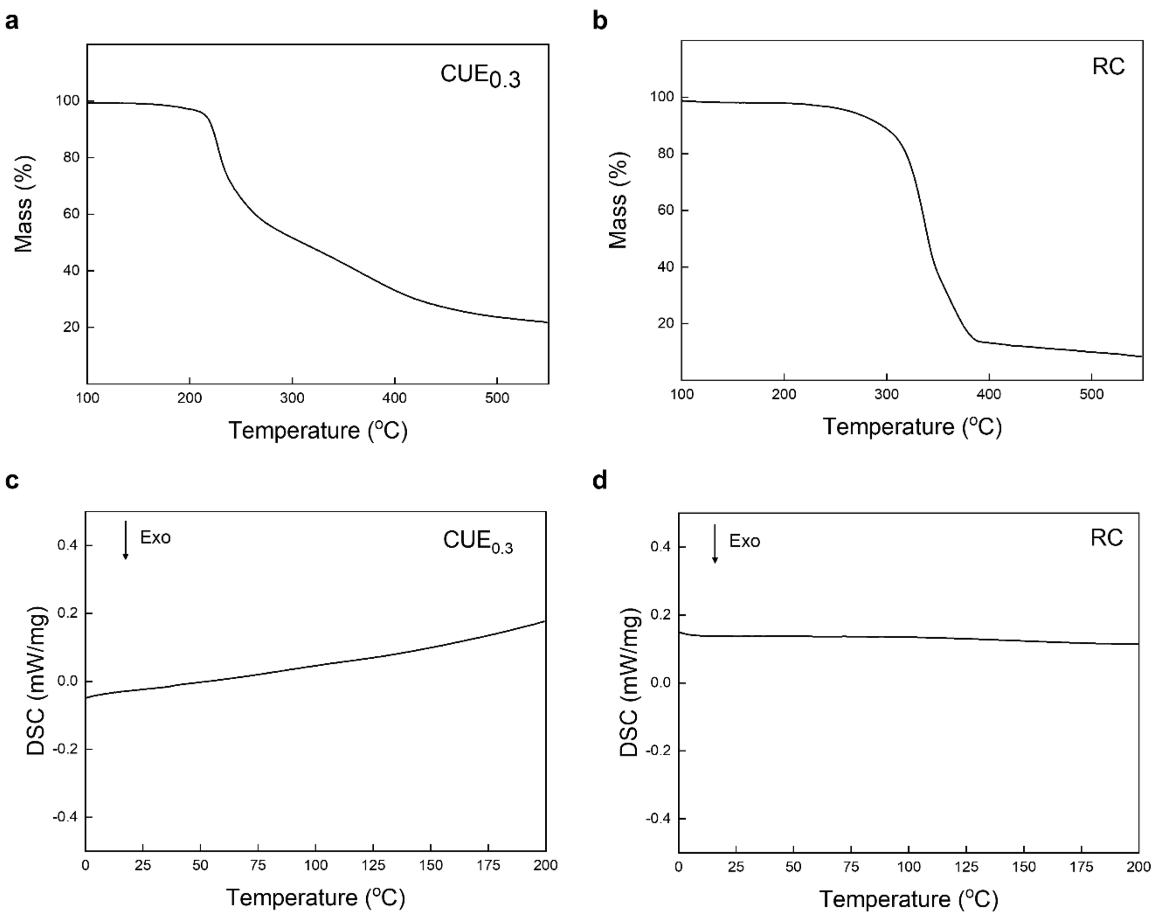

Figure S19.

TGA curves of as-prepared **(a)** $CUE_{0.3}$ and **(b)** RC membranes. DSC curves of as-prepared **(c)** $CUE_{0.3}$ and **(d)** RC membranes.

Table S1.

Luminous transmittance and haze of $CUE_{0.3}$ and RC membranes

Samples	Light transmittance (%)	Haze (%)
$CUE_{0.3}$	94.8 ± 0.2	29.7 ± 0.4
RC	95.1 ± 0.3	37.0 ± 0.6

Table S2.

SFE results of $CUE_{0.3}$ and RC membranes

Samples	Water contact angle (°)	Diiodomethane contact angle (°)	Disperse SFE (mN/m)	Polar SFE (mN/m)
$CUE_{0.3}$	88.07 ± 1.29	58.53 ± 0.31	32.39 ± 0.58	2.97 ± 0.40
RC	86.43 ± 2.48	63.69 ± 0.67	26.45 ± 0.39	4.21 ± 0.91

Table S3.

Calculated results of electron density and H-bond energy between $CUE_{0.3}$ and water

Location of H-bonds	Electron density at BCP (a. u.)	The fitted bond energy of H-bonds (kcal/mol)
H110……O19	0.02350	-4.50
H65……O108	0.02862	-5.64
H112……O47	0.00926	-1.32
H115……O21	0.01621	-2.87
H116……O35	0.02550	-4.95
H118……O10	0.01667	-2.98
H119……O6	0.01955	-3.62

Table S4.

Equilibrium strain of hydrous $CUE_{0.3}$ and RC membranes based on the consecutive plasticity mechano-creep experiments

Samples	Equilibrium strain (%)			
	**Stress of 2.5 MPa**	**Stress of 6.5 MPa**	**Stress of 10.5 MPa**	**Stress of 14.5 MPa**
$CUE_{0.3}$	0.6 ± 0.1	2.0 ± 0.3	5.1 ± 1.0	10.3 ± 1.8
RC	0.9 ± 0.2	4.7 ± 0.1	9.0 ± 0.1	14.2 ± 0.4

Table S5.

Residual stress of hydrous $CUE_{0.3}$ and RC membranes based on the consecutive plasticity mechano-stress relaxation experiments

**Samples**	**Residual Stress (%)**			
	**Strain of 1%**	**Strain of 3%**	**Strain of 5%**	**Strain of 7%**
$CUE_{0.3}$	51.7	49.2	49.3	49.1
RC	37.5	36.9	39.5	44.3

Reference

1. Sadeghifar, H.; Venditti, R.; Jur, J.; Gorga, R. E.; Pawlak, J. J., Cellulose-Lignin Biodegradable and Flexible UV Protection Film. *ACS Sustainable Chemistry & Engineering* **2017,** *5* (1), 625-631.
2. Samaranayake, G.; Glasser, W. G., Cellulose derivatives with low DS. I. A novel acylation system. *Carbohydrate Polymers* **1993,** *22* (1), 1-7.
3. Standard, A., ASTM (D882-02) Standard test method for tensile properties of thin plastic sheeting. West Conshohocken, PA. **2002**.
4. Neese, F., Software update: The ORCA program system—Version 5.0. *Wiley Interdisciplinary Reviews: Computational Molecular Science* **2022,** *12* (5), e1606.
5. Grimme, S.; Hansen, A.; Ehlert, S.; Mewes, J.-M., r2SCAN-3c: A “Swiss army knife” composite electronic-structure method. *The Journal of Chemical Physics* **2021,** *154* (6), 064103.
6. Zheng, J.; Xu, X.; Truhlar, D. G., Minimally augmented Karlsruhe basis sets. *Theoretical Chemistry Accounts* **2011,** *128*, 295-305.
7. Grimme, S.; Ehrlich, S.; Goerigk, L., Effect of the damping function in dispersion corrected density functional theory. *Journal of Computational Chemistry* **2011,** *32* (7), 1456-1465.
8. Lu, T.; Chen, F., Multiwfn: A multifunctional wavefunction analyzer. *Journal of Computational Chemistry* **2012,** *33* (5), 580-592.
9. Emamian, S.; Lu, T.; Kruse, H.; Emamian, H., Exploring nature and predicting strength of hydrogen bonds: A correlation analysis between atoms-in-molecules descriptors, binding energies, and energy components of symmetry-adapted perturbation theory. *Journal of Computational Chemistry* **2019,** *40* (32), 2868-2881.
10. Kulasinski, K.; Keten, S.; Churakov, S. V.; Derome, D.; Carmeliet, J., A comparative molecular dynamics study of crystalline, paracrystalline and amorphous states of cellulose. *Cellulose* **2014,** *21*, 1103-1116.
11. Chenoweth, K.; Van Duin, A. C.; Goddard, W. A., ReaxFF reactive force field for molecular dynamics simulations of hydrocarbon oxidation. *The Journal of Physical Chemistry A* **2008,** *112* (5), 1040-1053.
12. Plimpton, S., Fast parallel algorithms for short-range molecular dynamics. *Journal of Computational Physics* **1995,** *117* (1), 1-19.
13. Strachan, A.; van Duin, A. C.; Chakraborty, D.; Dasgupta, S.; Goddard III, W. A., Shock waves in high-energy materials: The initial chemical events in nitramine RDX. *Physical Review Letters* **2003,** *91* (9), 098301.
14. Sun, H.; Mumby, S. J.; Maple, J. R.; Hagler, A. T., An ab Initio CFF93 All-Atom Force Field for Polycarbonates. *Journal of the American Chemical Society* **1994,** *116* (7), 2978-2987.
15. Gowers, R. J.; Carbone, P., A multiscale approach to model hydrogen bonding: The case of polyamide. *The Journal of Chemical Physics* **2015,** *142* (22), 224907.
16. Meng, X.; Matson, J. B.; Edgar, K. J., Olefin cross-metathesis as a source of polysaccharide derivatives: Cellulose ω-carboxyalkanoates. *Biomacromolecules* **2014,** *15* (1), 177-187.
17. Dais, P.; Perlin, A. S., Assignment of the O-acetyl carbonyl carbon atoms of cellulose triacetate via 2D, long-range, proton-carbon chemical-shift-correlation data. *Carbohydrate Research* **1988,** *181*, 233-235.

18. Geissler, A.; Chen, L.; Zhang, K.; Bonaccurso, E.; Biesalski, M., Superhydrophobic surfaces fabricated from nano-and microstructured cellulose stearoyl esters. *Chemical Communications* **2013,** *49* (43), 4962-4964.
19. Vaca-Garcia, C.; Borredon, M.-E.; Gaseta, A., Determination of the degree of substitution (DS) of mixed cellulose esters by elemental analysis. *Cellulose* **2001,** *8*, 225-231.
20. Rubinstein, M.; Colby, R. H., *Polymer physics*. Oxford university press New York: 2003; Vol. 23.
21. Yakimets, I.; Paes, S. S.; Wellner, N.; Smith, A. C.; Wilson, R. H.; Mitchell, J. R., Effect of water content on the structural reorganization and elastic properties of biopolymer films: a comparative study. *Biomacromolecules* **2007,** *8* (5), 1710-1722.
